# Effects of exenatide and open-label SGLT2 inhibitor treatment, given in parallel or sequentially, on mortality and cardiovascular and renal outcomes in type 2 diabetes: insights from the EXSCEL trial

**DOI:** 10.1186/s12933-019-0942-x

**Published:** 2019-10-22

**Authors:** Lindsay E. Clegg, Robert C. Penland, Srinivas Bachina, David W. Boulton, Marcus Thuresson, Hiddo J. L. Heerspink, Stephanie Gustavson, C. David Sjöström, James A. Ruggles, Adrian F. Hernandez, John B. Buse, Robert J. Mentz, Rury R. Holman

**Affiliations:** 1 Clinical Pharmacology and Safety Sciences, R&D, AstraZeneca, 1 MedImmune Way, Gaithersburg, MD 20878 USA; 2 Clinical Pharmacology and Safety Sciences, R&D, AstraZeneca, Boston, USA; 3 Statisticon AB, Uppsala, Sweden; 4 Department of Clinical Pharmacy and Pharmacology, University of Groningen, University Medical Center Groningen, Groningen, The Netherlands; 5 BioPharmaceuticals R&D, AstraZeneca, Gaithersburg, MD USA; 6 BioPharmaceuticals R&D, AstraZeneca, Gothenburg, Sweden; 7 BioPharmaceuticals, AstraZeneca, Wilmington, DE USA; 8 Duke University and Duke Clinical Research Institute, Duke University School of Medicine, Durham, NC USA; 9 University of North Carolina School of Medicine, Chapel Hill, NC USA; 10 Diabetes Trials Unit, University of Oxford, Oxford, UK

**Keywords:** SGLT2 inhibitor, GLP-1 receptor agonist, Exenatide, Cardiovascular outcomes, eGFR slope, Type 2 diabetes mellitus, Combination therapy, Propensity score matching

## Abstract

**Background:**

Sodium-glucose cotransporter-2 inhibitors (SGLT2i) and glucagon-like peptide-1 receptor agonists (GLP-1 RA) improve cardiovascular and renal outcomes in patients with type 2 diabetes through distinct mechanisms. However, evidence on clinical outcomes in patients treated with both GLP-1 RA and SGLT2i is lacking. We aim to provide insight into the effects of open-label SGLT2i use in parallel with or shortly after once-weekly GLP-1 RA exenatide (EQW) on cardiorenal outcomes.

**Methods:**

In the EXSCEL cardiovascular outcomes trial EQW arm, SGLT2i drop-in occurred in 8.7% of participants. These EQW+SGLT2i users were propensity-matched to: (1) placebo-arm participants not taking SGLT2i (n = 572 per group); and to (2) EQW-arm participants not taking SGLT2i (n = 575), based on their last measured characteristics before SGLT2i initiation, and equivalent study visit in comparator groups. Time-to-first major adverse cardiovascular event (MACE) and all-cause mortality (ACM) were compared using Cox regression analyses. eGFR slopes were quantified using mixed model repeated measurement analyses.

**Results:**

In adjusted analyses, the risk for MACE with combination EQW+SGLT2i use was numerically lower compared with both placebo (adjusted hazard ratio 0.68, 95% CI 0.39–1.17) and EQW alone (0.85, 0.48–1.49). Risk of ACM was nominally significantly reduced compared with placebo (0.38, 0.16–0.90) and compared with EQW (0.41, 0.17–0.95). Combination EQW+SGLT2i use also nominally significantly improved estimated eGFR slope compared with placebo (+ 1.94, 95% CI 0.94–2.94 mL/min/1.73 m^2^/year) and EQW alone (+ 2.38, 1.40–3.35 mL/min/1.73 m^2^/year).

**Conclusions:**

This *post hoc* analysis supports the hypothesis that combinatorial EQW and SGLT2i therapy may provide benefit on cardiovascular outcomes and mortality.

*Trial registration* Clinicaltrials.gov, Identifying number: NCT01144338, Date of registration: June 15, 2010.

## Background

Members of both the glucagon-like peptide-1 receptor agonist (GLP-1 RA) and sodium-glucose cotransporter-2 inhibitor (SGLT2i) classes of anti-hyperglycemic drugs have demonstrated cardio- and reno-protective effects in cardiovascular outcomes trials (CVOTs) conducted in patients with type 2 diabetes mellitus. While the study populations, designs, and results of individual trials have varied, meta-analyses suggest that both classes reduce major adverse cardiovascular events (MACE) in participants with established atherosclerotic cardiovascular disease (ASCVD) [1–4]. These meta-analyses additionally show that SGLT2i reduce risk of hospitalization for heart failure and the risk of worsening estimated glomerular filtration rate (eGFR) or end-stage renal disease, regardless of ASCVD status [1–3].

Many patients require multiple anti-hyperglycemic agents to manage their diabetes. Given the largely distinct mechanisms, beyond glycemic control, through which GLP-1 RA and SGLT2i are hypothesized to exert their cardiorenal effects, there is considerable interest in how the combination of these two classes would effect long-term outcomes [5, 6]. The 104 week DURATION-8, 24 week AWARD-10, and 30 week SUSTAIN 9 trials demonstrated that simultaneous initiation of GLP-1 RA exenatide and SGLT2i dapagliflozin, or addition of GLP-1 RAs dulaglutide and semaglutide to open-label SGLT2i, durably improved glycemic control and cardiovascular risk factors without increasing the risk of hypoglycemia [7–10]. However, no clinical trial or real world evidence on long-term cardiovascular outcomes, mortality, and renal disease progression with combination GLP-1 RA and SGLT2i treatment have been reported to date.

The EXenatide Study of Cardiovascular Event Lowering (EXSCEL) was a randomized, placebo-controlled, global pragmatic clinical trial designed to assess the effect of subcutaneous once-weekly GLP-1 RA exenatide (EQW) 2 mg on cardiovascular outcomes in 14,752 participants with type 2 diabetes mellitus and a range of cardiovascular risk [11]. Potential participants were permitted to take up to three oral anti-hyperglycemic drugs, or insulin in combination with up to two oral anti-hyperglycemic agents, as part of usual care for their diabetes management. During the course of EXSCEL, three SGLT2i were approved and marketed.

This *posthoc* analysis of EXSCEL leveraged the pool of participants taking open label SGLT2i in addition to study drug to quantify the impact of this EQW+SGLT2i combination on cardiorenal outcomes, as compared with both: (1) treatment with neither EQW nor an SGLT2i, and (2) treatment with EQW but not an SGLT2i, on top of standard of care.

## Methods

### Population and SGLT2i usage

EXSCEL (NCT01144338) enrolled 14,752 patients in 35 countries between June 2010 and September 2015. The primary results, study design and baseline characteristics have been published [11–13]. Briefly, inclusion criteria included a hemoglobin A1c (HbA_1c_) of 6.5% to 10%, and any level of cardiovascular risk, targeting ~ 70% of participants with a previous cardiovascular event. Participants were excluded if they were < 18 years old, had type 1 diabetes, ≥ 2 episodes of severe hypoglycemia in the previous 12 months, an estimated glomerular filtration rate (eGFR) < 30 mL/min/1.73 m^2^, or previous pancreatitis.

All EXSCEL participants were included in this analysis except for 786 placebo arm participants that took open-label SGLT2i. The 33 EXSCEL participants who never received study drug and a further 635 participants missing required covariates were excluded from propensity matching (Additional file 1: Figure S1).

Information on SGLT2i use was collected at each 6-monthly study visit [11]. As precise dates for concomitant medication initiation and cessation were not itemized, we assumed SGLT2i initiation at the first study visit that recorded its use [14]. SGLT2i exposure time was calculated as the interval from the first visit with known usage to the last study visit with SGLT2i use recorded, regardless of gaps or switching of SGLT2i type. No lower bound on exposure was imposed. We assumed no SGLT2i use when information was missing; SGLT2i usage data was not collected before May 2013, 6 months after the first market approval of an SGLT2i. Due to the limited size of the available data, subjects were eligible for inclusion in the EQW+SGLT2i cohorts whether they discontinued study drug before SGLT2i initiation or not.

### Endpoints

This *posthoc* analysis examined multiple prespecified time-to-event EXSCEL endpoints: (1) first adjudicated composite of a three-point major adverse cardiovascular event (MACE), defined as cardiovascular death, nonfatal myocardial infarction or nonfatal stroke, the primary endpoint of the trial; (2) all-cause mortality (ACM); (3) cardiovascular death; and (4) serious hypoglycemia, defined as hypoglycemia requiring third-party assistance. Change over time in MDRD eGFR was examined to quantify renal disease progression. Exploratory time-to-event analyses were performed for: (1) hospitalization for heart failure (hHF); (2) a composite of hospitalization for heart failure and cardiovascular death; (3) nonfatal myocardial infarction; (4) nonfatal stroke; (5) a composite of a persistent 40% reduction in eGFR, renal dialysis, or renal transplant (“Renal_1”), (6) a composite of Renal_1 plus new macroalbuminuria (“Renal_2”); and (7) amputation.

### Propensity matching

Propensity matching was performed using the same protocol as outlined in [14], designed to balance medical history, demographics, laboratory measurements, medication use, and follow-up time. Participants taking SGLT2i in the EQW arm were matched at the first known SGLT2i use to: (1) participants in the placebo arm that did not take SGLT2i (“Placebo comparison”); and (2) participants in the EQW arm that did not take SGLT2i (“Exenatide comparison”), based on covariates at the comparable study visit. No comparison was made to placebo+SGLT2i users because a large comparator group is necessary to achieve a well-matched set of cohorts, and both the EQW+SGLT2i and placebo+SGLT2i groups in EXSCEL were moderately-sized. Matching was performed first for the baseline visit, and then subsequently for each study visit without replacement of participants already matched. Propensity scores were calculated across all available participants and visits using a generalized linear model, and matching was performed via the nearest neighbor approach, using this score and logit distance calculated at each visit. A caliper of 0.1 and a 1:1 matching ratio were used in the R package MatchIt [15]. A matched set of cohorts were accepted if the post-matching difference between treatment groups for every covariate was less than 0.1 standardized difference.

The nearest neighbor approach matches participants in a random order, resulting in changes to the cohorts if matching is repeated. To prevent selection bias, the first set of cohorts generated that met the acceptance criteria were selected for this analysis. To assess the robustness of the results to variability in matching, we repeated matching 5000 times for each comparison, and compared the distributions of estimated hazard ratios in the accepted cohorts to those in the primary analysis. We also generated cohorts matched to only subjects taking exenatide and SGLT2i simultaneously, and performed a sensitivity analysis where we censored subjects in the primary analysis at initiation of open-label GLP-1 RA (in violation of study protocol) to further probe the impact of variation in drug usage patterns within our cohorts.

Covariates used for propensity matching were: age, sex, ethnicity, smoking status (trial baseline), race, region, duration of diabetes, history of heart failure, history of prior cardiovascular disease (CVD), microalbuminuria, macroalbuminuria, BMI, eGFR, systolic blood pressure, HbA_1c_, total cholesterol, and use of renin-angiotensin-aldosterone system inhibitors, thiazolidinediones, metformin, dipeptidyl peptidase-4 inhibitors, and insulin. All covariates were evaluated based on the last information *prior* to the first known SGLT2i usage (or comparable visit in controls). For participants on SGLT2i prior to enrollment, baseline characteristics were used. Prior CVD was defined, per EXSCEL protocol, as major clinical manifestations of coronary artery disease, atherosclerotic peripheral artery disease, or ischemic cerebrovascular disease, and was updated from trial baseline status based on recorded incidence of MACE, peripheral artery or vascular disease, coronary catheterization, angioplasty or stenting, coronary artery bypass, or percutaneous coronary intervention. Similarly, history of heart failure, age, and duration of diabetes were updated from trial baseline to reflect recorded hHF and time in trial, respectively.

As EXSCEL collected only local laboratory measurements, non-physiologically-reasonable outliers were capped prior to matching using the following cut-offs: BMI > 60 kg/m^2^, eGFR > 250 mL/min/1.73 m^2^, HbA_1c_ > 15% (140 mmol/mol), and total cholesterol > 15 mmol/L. These cutoffs were set prior to analysis based on expected physiological ranges, and we confirmed that only a small number of all trial measurements were capped: 164 BMI (0.2%), 20 eGFR (0.02%), 49 HbA_1c_ (0.06%), and 15 cholesterol (0.02%).

### Time-to-event analyses

Hazard ratios (HR) for time-to-first-event analyses in both sets of propensity-matched cohorts were calculated via Cox proportional hazards regression. Analyses were performed with treatment as the sole exploratory variable (unadjusted), and adjusted for: duration of diabetes, age, sex, history of CVD, prior heart failure, prior microalbuminuria, prior macroalbuminuria, eGFR, and HbA_1c_, to provide a “doubly robust” estimator with lower risk of bias than would be obtained via propensity matching or adjustment alone [16]. The number of adjustment covariates was constrained because of the limited size of the matched cohorts [17].

Follow-up time began at matching (SGLT2i initiation or equivalent study visit in controls), and continued until the end of trial follow-up, regardless of study drug or SGLT2i discontinuation. Participants with an event before matching were censored at time zero for analysis of that endpoint. For composite endpoints, censoring occurred at the last information on the earliest censored component. For the renal composites, renal dialysis or transplant events occurring within 30 days of the last eGFR measurement were included.

Only participants with at least two post-matching eGFR measurements were included in renal time-to-event analyses (Additional file 1: Table S3). Persistent 40% eGFR reduction was defined as two sequential post-matching eGFR measurements ≤ 60% of the last pre-match eGFR measurement, with eGFR values over 250 mL/min/1.73 m^2^ excluded. eGFR was calculated centrally using the MDRD formula, based on local, site-reported serum creatinine measurements [18].

Endpoints were analyzed in both comparisons, but no direct comparisons were made between the two sets of matched cohorts. Owing to the *posthoc* nature of this analysis, all reported p-values are nominal and no multiple test corrections were performed. A p-value < 0.05 was considered nominally significant.

### eGFR slope

eGFR slope vs. time was analyzed in both sets of propensity-matched cohorts using a mixed-model repeated measures (MMRM) analysis to estimate the overall treatment effect on eGFR slope. MDRD-based eGFR was the dependent variable, with time (scheduled visit window) and baseline eGFR as linear covariates, treatment arm and visit-by-treatment interaction as fixed effects, and patient as a random effect.

### Software

Data was prepared in SAS 9.4, and all analyses were performed in R version 3.4.0 [19].

## Results

### Full population characteristics and drug use

**Fig. 1:**
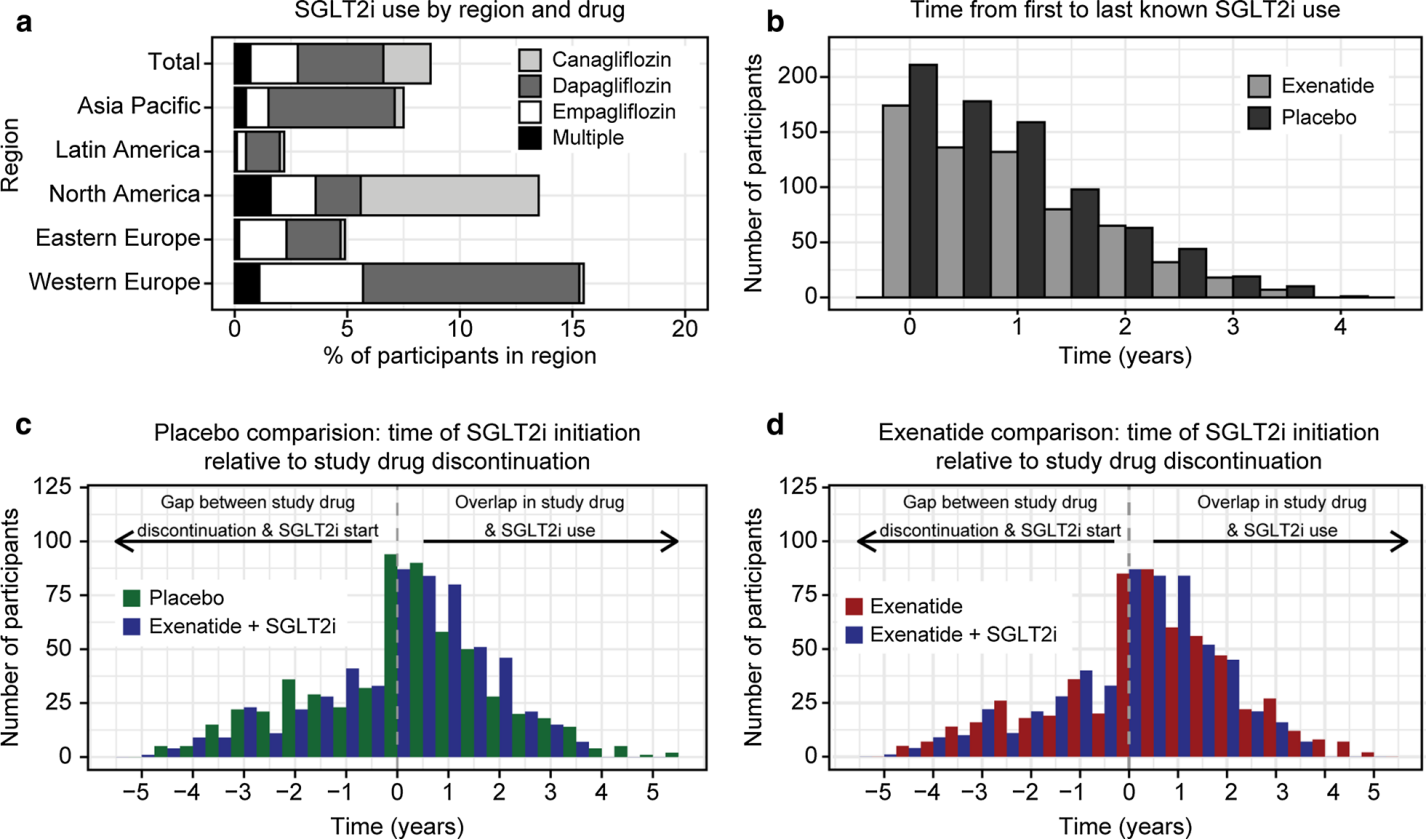
SGLT2i usage in EXSCEL. **a** Percentage of exenatide arm participants taking SGLT2i at some point, by drug and by region. “Multiple” indicates use of more than one SGLT2i during the trial. **b** Histogram of time from first to last known SGLT2i use in the EXSCEL exenatide QW (light gray) and placebo (dark gray) arms. Note that, given lack of precise start/stop dates, this estimate of length of SGLT2i exposure represents a lower bound. **c**, **d** Time of SGLT2i initiation or matching relative to discontinuation of EQW or placebo in the propensity-matched cohorts. Blue: combination EQW+SGLT2i cohort; green: placebo cohort; red: EQW cohort

During the course of EXSCEL, 645 EQW arm participants used an SGLT2i at some point, with the highest SGLT2i use in Western Europe and North America (Fig. 1a). Compared with non-SGLT2i users, these participants were more likely to be male (68% vs 61%), white (84% vs. 75%), have less history of CV disease (62% vs. 74%) and heart failure (9.1% vs. 17%) at trial baseline, and have more history of albuminuria (23% vs. 16%) (Additional file 1: Table S2) [12]. This group also had higher HbA1c (8.3% vs. 8.1% (67 mmol/mol vs. 65 mmol/mol)) and eGFR (85 vs. 76 mL/min/1.73 m^2^), and took more anti-hyperglycemic agents aside from GLP-1 RA and SGLT2i (1.7 vs. 1.3) than non-SGLT2i users in EXSCEL (Additional file 1: Table S2).

Of the three SGLT2i taken by EXSCEL participants, dapagliflozin was the most commonly used (Additional file 1: Table S1). Median time of first known SGLT2i use in the EQW arm was 2.7 years (interquartile range (IQR) 1.5–3.8 years) (Additional file 1: Figure S2A), and median time from first to last known SGLT2i use was 9.3 months (IQR 2.5–18.1 months) (Fig. 1b).

#### Propensity matching

Of the 623 SGLT2i users in the EQW arm with all required covariates (Additional file 1: Figure S1), 572 were matched in the placebo comparison (92%), and 575 were matched in the EQW comparison (92%), with all covariates having an imbalance of less than 0.1 standardized difference in the accepted sets of cohorts Additional file 1: Figures S3A, B), and similar propensity score distributions between cohorts (Additional file 1: Figure S4). These cohorts (Table 1) were generally similar to the overall distribution of SGLT2i users in the EQW arm (Additional file 1: Table S2), though, importantly, different than the overall population of EXSCEL [12]. 560 of the participants in the EQW+SGLT2i cohorts were identical between the two comparisons. Characteristics in the separately matched “placebo” and “EQW” comparator cohorts were generally similar, but subjects in the “EQW” cohort were older, had a longer duration of diabetes, and were less likely to be Hispanic (Additional file 1: Figure S3C), limiting validity of direct comparisons between these groups. Median follow-up time for ACM in the placebo comparison was 13.3 months (IQR 6.5–23.1) for the combination cohort and 14.2 months (IQR 4.5–27.0) for the placebo cohort. In the EQW comparison, median follow-up was 13.3 months (IQR 6.5–23.0) in the combination cohort and 15.1 months (IQR 6.0–26.7) in the exenatide cohort.

**Table 1:** Clinical characteristics of propensity-matched cohorts at time of matching

	Placebo comparison		Exenatide comparison	
	Placebo, No SGLT2i	Exenatide QW + SGLT2i	Exenatide, No SGLT2i	Exenatide QW + SGLT2i
Participants, n	572	572	575	575
Sex, male	380 (66%)	391 (68%)	399 (69%)	395 (69%)
Age, years	62 (10)	62 (9)	63 (10)	62 (9)
Race				
White	486 (85%)	487 (85%)	495 (86%)	488 (85%)
Black	14 (2.4%)	20 (3.5%)	24 (4.2%)	22 (3.8%)
Asian	52 (9.1%)	50 (8.7%)	44 (7.7%)	50 (8.7%)
Other/unknown	20 (3.5%)	15 (2.6%)	12 (2.1%)	15 (2.6%)
Region				
North America	213 (37%)	212 (37%)	210 (37%)	215 (38%)
Latin America	27 (4.7%)	27 (4.7%)	25 (4.3%)	26 (4.5%)
Asia Pacific	53 (9.3%)	54 (9.4%)	50 (8.7%)	54 (9.4%)
Western Europe	200 (35%)	182 (32%)	190 (33%)	186 (32%)
Eastern Europe	79 (14%)	97 (17%)	100 (17%)	94 (16%)
Ethnicity, Hispanic	47 (8.2%)	33 (5.8%)	32 (5.6%)	33 (5.7%)
Duration of diabetes, years	16 (8)	16 (8)	17 (9)	16 (8)
History of CVD (CAD, PAD, or stroke)	377 (66%)	379 (66%)	395 (69%)	378 (66%)
History of heart failure	64 (11%)	62 (11%)	65 (11%)	63 (11%)
History of retinopathy	99 (17%)	108 (19%)	108 (19%)	109 (19%)
History of micro- or macro-albuminuria	172 (30%)	159 (28%)	164 (29%)	160 (28%)
Microalbuminuria	147 (26%)	139 (24%)	134 (23%)	140 (24%)
Macroalbuminuria	31 (5.4%)	28 (4.9%)	34 (5.9%)	28 (4.9%)
Systolic blood pressure, mmHg	133.6 (16.2)	133.4 (15.4)	133.1 (15.7)	133.4 (15.5)
Diastolic blood pressure, mmHg	76.6 (10.4)	77.5 (10.0)	77.3 (10.1)	77.5 (10.0)
BMI, kg/m^2^	33.9 (6.9)	34.1 (6.3)	34.1 (6.5)	34.1 (6.3)
HbA_1c_, %	8.3 (1.5)	8.2 (1.2)	8.2 (1.6)	8.2 (1.2)
Cholesterol, mmol/L	4.2 (1.2)	4.2 (1.2)	4.2 (1.2)	4.2 (1.2)
LDL, mmol/L	2.3 (1.0)	2.2 (1.0)	2.2 (0.9)	2.2 (1.0)
HDL, mmol/L	1.1 (0.3)	1.1 (0.3)	1.1 (0.3)	1.1 (0.3)
UACR (median, IQR), g/mol	2.2 [0.9,6.6]	1.4 [0.5,4.4]	1.9 [0.6,5.0]	1.4 [0.5,4.2]
Hemoglobin, g/L	137.3 (15.6)	140.2 (16.3)	137.0 (15.4)	140.1 (16.4)
eGFR, mL/min/1.73 m^2^	79.7 (26.4)	81.1 (22.0)	79.6 (25.8)	81.1 (22.2)
eGFR<60 mL/min/1.73 m^2^	130 (23%)	93 (16%)	132 (23%)	94 (16%)
eGFR<45 mL/min/1.73 m^2^	46 (8.0%)	18 (3.1%)	42 (7.3%)	19 (3.3%)
Smoking				
Never	83 (15%)	77 (13%)	67 (12%)	75 (13%)
Past	234 (41%)	231 (40%)	258 (45%)	235 (41%)
Current	255 (45%)	264 (46%)	250 (43%)	265 (46%)
Classes of diabetes medications (n)^a^	1.6 (0.9)	1.5 (0.9)	1.5 (0.9)	1.5 (0.9)
RAASi	458 (80%)	461 (81%)	471 (82%)	463 (81%)
Other antihypertensives	325 (57%)	353 (62%)	336 (58%)	353 (61%)
Statins	434 (76%)	460 (80%)	433 (75%)	462 (80%)
Diuretics	247 (43%)	240 (42%)	266 (46%)	238 (41%)
Insulin	312 (55%)	324 (57%)	321 (56%)	323 (56%)
Metformin	472 (83%)	482 (84%)	480 (83%)	481 (84%)
TZD	22 (3.8%)	29 (5.1%)	31 (5.4%)	32 (5.6%)
DPP-4i	192 (34%)	176 (31%)	186 (32%)	172 (30%)
Sulfonylureas	215 (38%)	190 (33%)	182 (32%)	189 (33%)

Continuous metrics are reported as mean (SD). Categorical metrics are reported as n (%)

*BMI* body mass index, *CAD* coronary artery disease, *CVD* cardiovascular disease, *DPP*-*4i* dipeptidyl peptidase-4 inhibitors, *eGFR* estimated glomerular filtration rate, *GLP1*-*RA* glucagon‑like peptide‑1 receptor agonists, *HbA*_*1c*_ glycated hemoglobin, *HDL* high-density lipoproteins, *LDL* low-density lipoproteins, *PAD* peripheral artery disease, *RAASi* renin-angiotensin-aldosterone system inhibitors, *SD* standard deviation, *SGLT2i* sodium-glucose co-transporter-2 inhibitors, *TZD* thiazolidinediones, *UACR* urinary albumin-to-creatine ratio

^a^Classes of anti-hyperglycemic agents included: biguanides, sulfonylureas, meglitinides, DPP-4i, and TZD. Insulin, SGLT2i, and GLP1-RA (excluded by study protocol) are not included

Median study drug exposure (from trial baseline) in the propensity-matched cohorts ranged from 29.7 to 38.4 months (Additional file 1: Figure S2C, D). Median time from first to last known SGLT2i use was 9.8 months (IQR 3.1–18.8) in both comparisons. Median time from SGLT2i initiation/matching to study drug discontinuation in the placebo comparison was 3.2 months [IQR from 12.2 months before matching to 14.8 months after matching] in the placebo cohort and 4.7 months [9.7 months before to 14.8 months after] in the combination cohort (Fig. 1c). In the exenatide comparison, median time from SGLT2i initiation/matching to study drug discontinuation was 5.6 months [IQR 7.9 months before to 19.0 months after] in the EQW cohort and 4.9 months [IQR 9.2 months before to 14.8 months after] in the combination cohort (Fig. 1d). In total, 344 out of 572 participants (60%) in the placebo comparison and 348 of 575 participants (61%) in the EQW comparison started SGLT2i before EQW discontinuation.

#### Time-to-event analyses

**Fig. 2:**
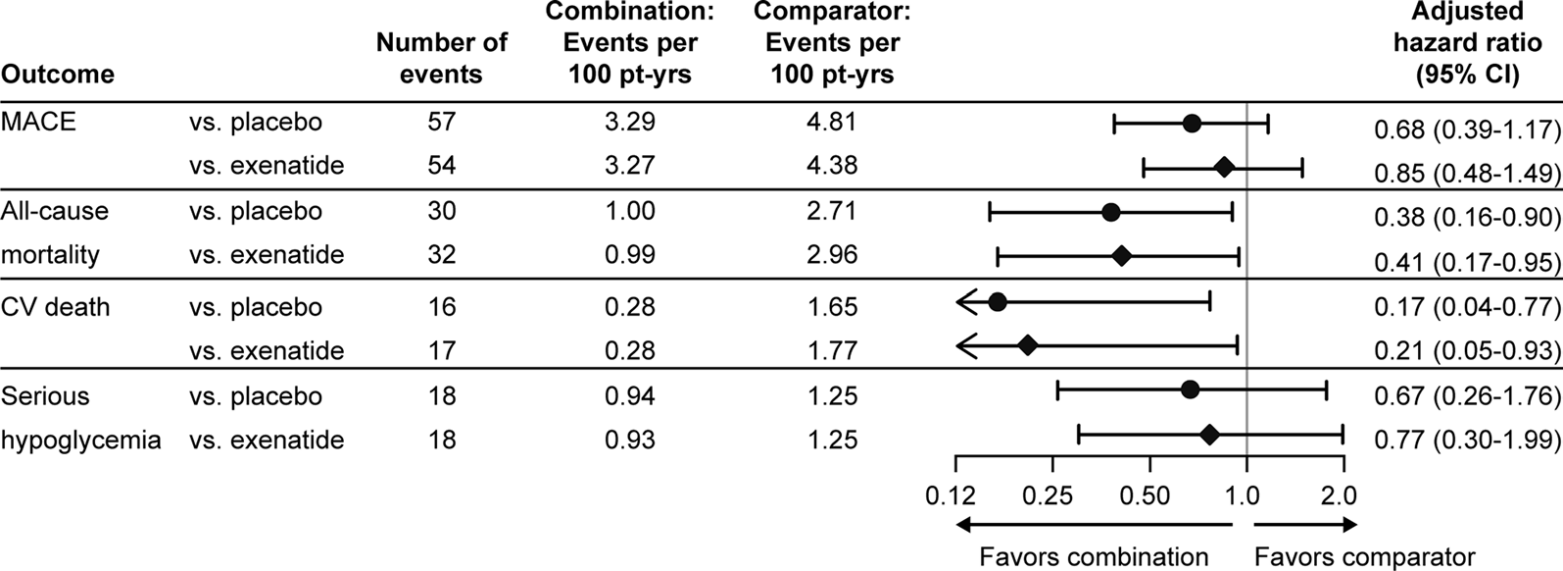
Cardiovascular, mortality, and safety outcomes with combination exenatide QW+SGLT2i in the propensity-matched cohorts. Additional details are found in Additional file 1: Tables S3, S4, and Kaplan–Meier curves in Additional file 1: Figures S5–S9. Hazard ratio adjusted for age, duration of diabetes, prior cardiovascular disease, heart failure, sex, microalbuminuria, macroalbuminuria, eGFR, and HbA1c, all evaluated at first known SGLT2i usage or equivalent visit in comparator groups. MACE: major adverse cardiovascular events; CV: cardiovascular; pt-yrs: participant-years

Cox proportional hazard models were used to estimate hazard ratios for MACE, ACM, CV death, and serious hypoglycemia in each of the two sets of cohorts. In both comparisons, the hazard ratio for MACE with EQW+SGLT2i was non-significantly decreased compared with placebo (adjusted HR (95% CI) 0.68 (0.39–1.17)), and compared with EQW (0.85 (0.48–1.49), Fig. 2). This reduction was driven by a nominally significant decrease in risk of CV death (aHR 0.17 (0.04–0.77) compared with placebo and 0.21 (0.05–0.93) compared with EQW); nonfatal MI and nonfatal stroke were unchanged (Additional file 1: Tables S3 and S4). All-cause mortality was also reduced (aHR vs. placebo of 0.38 (0.16–0.90) and vs. EQW of 0.41 (0.17–0.95)), with no increase in risk of serious hypoglycemia (Fig. 2). Kaplan–Meier curves are shown in Additional file 1: Figures S5–S9.

In the exploratory analysis, the composite of hHF and CV death was nominally significantly reduced in both comparisons (Additional file 1: Tables S3 and S4), driven by CV death, while hHF was not reduced. Event numbers were small for both renal composites, with non-significant reductions in the estimated hazard ratios (Additional file 1: Tables S3 and S4). Amputations were rare (5 events total) and not significantly different in either comparison (Additional file 1: Tables S3 and S4).

In the nearest neighbor method, re-matching changes the composition of the comparator cohorts, and the resultant event rates. To account for this random effect, we repeated the propensity matching procedure 5000 times, generating approximately 2000 accepted sets of cohorts for each comparison. Comparing the distributions of hazard ratio estimates in these sets to the primary case reported here confirms that those for MACE, ACM, and CV death are near the centers of the respective distributions (Additional file 1: Figure S10A, B). The estimate for hHF in the placebo comparison is higher than most estimates, likely due to random chance and low event numbers (Additional file 1: Figure S10C), with most runs suggesting a numerically lower hHF risk with EQW+SGLT2i in both comparisons.

Next, we asked whether the observed effects were similar in only subjects taking EQW and SGLT2i simultaneously. In these smaller cohorts (n = 336–340 per cohort), no MACE benefit was observed, but trends for reduced ACM and CV death were consistent with the primary analysis (Additional file 1: Tables S5 and S6). Additionally, censoring of subjects initiating open-label GLP-1 RA had very little impact on estimated hazard ratios (Additional file 1: Tables S7 and S8).

#### eGFR slope

**Fig. 3:**
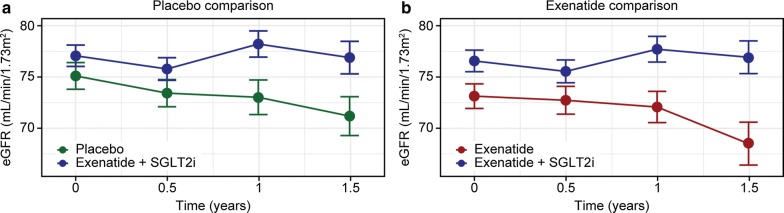
Geometric mean (+/− standard error) eGFR in the propensity-matched cohorts. **a** Placebo comparison. **b** Exenatide comparison. Time zero is the time of first visit with known SGLT2i use/matching. The small differences in eGFR at matching (t=0) also reflect restrictions on SGLT2i use in moderate renal impairment. eGFR slopes prior to matching are shown in Additional file 1: Figure S11. Blue: combination exenatide + SGLT2i cohorts; green: placebo cohort; red: exenatide cohort. eGFR, estimated glomerular filtration rate; SGLT2i, sodium-glucose co-transporter-2 inhibitor

Figure 3 shows geometric mean eGFR over time after matching in the two sets of propensity-matched cohorts. MMRM-estimated slopes in the placebo comparison were + 1.21 mL/min/1.73 m^2^/year (se 0.37) in the combination cohort and − 0.71 mL/min/1.73 m^2^/year (se 0.33) in the placebo cohort, resulting in an estimated treatment effect of + 1.94 mL/min/1.73 m^2^/year (95% CI 0.94–2.94, p < 0.001). Similarly, in the EQW comparison, the estimated slope was + 1.27 mL/min/1.73 m^2^/year (se 0.38) in the combination cohort, and − 1.11 mL/min/1.73 m^2^/year (se 0.32) in the EQW cohort, giving an estimated treatment effect of + 2.38 mL/min/1.73 m^2^/year (95% CI 1.40–3.35, p < 0.001).

## Discussion

This analysis provides the first clinical support for the hypothesis that combinatorial use of GLP-1 RA and SGLT2i therapies may provide additional benefit on adjudicated cardiovascular outcomes, mortality, and renal disease progression, compared with GLP-1 RA without SGLT2i, and to standard of care with neither GLP-1 RA nor SGLT2i (Table 2). The subset of EXSCEL participants using SGLT2i provide a modestly-sized but credible cohort with rigorously collected clinical outcomes. Our analysis demonstrated numerically lower MACE risk, driven by a nominally significant reduction in cardiovascular death, compared with both EQW alone and placebo. The reduction in ACM was also nominally significant in both comparisons, as was the improvement in eGFR slope, with no increase in risk of serious hypoglycemia. We did not have enough data to probe the contributions of each drug to the observed outcomes, or to compare to SGLT2i alone, and imbalances between the EQW alone and placebo cohorts warrant caution in comparing the two sets of cohorts side-by-side. Nonetheless, the trend across cardiovascular and mortality outcomes for a numerically larger risk reduction compared with placebo than compared with exenatide supports possible beneficial contributions from both drug classes. Conversely, the similar improvement in eGFR slope in both comparisons in our population suggests the observed eGFR slope improvement may be due primarily to SGLT2i contributions when used on top of GLP-1 RA, consistent with previous GLP-1 RA studies generally showing an effect more so on macroalbuminuria than on eGFR-based endpoints [4].

**Table 2:** Summary of key results

*Previous knowledge in the field*
Multiple GLP-1 RA and SGLT2i demonstrated benefit on cardiovascular outcomes, mortality, and/or renal disease progression
The mechanisms underlying these effects, while not fully understood, are largely distinct
Combination GLP-1 RA and SGLT2i treatment improves metabolic parameters and cardiovascular risk factors, but no data on cardiovascular events, mortality, or renal function decline is available
*New insights from this study*
Combination exenatide QW and SGLT2i numerically lowered the hazard ratio for MACE, driven by a significant reduction in cardiovascular death compared to exenatide alone or neither drug class
All-cause mortality risk decreased with the combination, compared to exenatide QW alone or placebo
SGLT2i-mediated eGFR slope improvement was consistent on top of placebo or exenatide QW treatment
This data supports the hypothesis that combination GLP-1 RA and SGLT2i may provide additional cardiovascular and mortality benefit to GLP-1 RA alone, without any increase in risk of hypoglycemia

In the full EXSCEL trial, the hazard ratio for MACE with EQW treatment was 0.91 (95% CI 0.83–1.00) [11], while several meta-analyses of the GLP-1 RA class estimated MACE hazard ratios from 0.87 to 0.90 [2–4, 20] for the class as a whole, with moderate evidence for intra-class heterogeneity. All three components of the MACE composite—cardiovascular death, non-fatal MI, and non-fatal stroke—appear to contribute to this outcome for the GLP-1 RA class [2, 3, 20]. While the majority of evidence supports this MACE benefit in subjects with ASCVD, there is some evidence that this benefit may extend to high-risk subjects without a previous CV event [4, 21]. In EXSCEL, the secondary end-point of ACM, while not formally tested due to the hierarchical nature of the statistical analysis plan, was nominally improved by EQW treatment (HR 0.86 (0.77–0.97) [13]. Meta-analyses suggest this mortality benefit is a class effect (hazard ratios 0.88–0.89) [4, 20], while no significant benefit on hHF or occurrence of serious hypoglycemia has been observed for the GLP-1 RA class [2, 3, 20]. In general GLP-1 RA trials point to a strong effect on new persistent macroalbuminuria [22–24], but there is limited evidence of improvement on eGFR-based endpoints: sustained eGFR decline of 40–50% for dulaglutide [23], and a small reduction in eGFR decline with liraglutide and dulaglutude in participants with moderate-to-severe CKD.

Trial design and study populations also varied across the SGLT2i class, with MACE hazard ratios in CVOTs of 0.86 to 0.93 [25–27]. Again, meta-analyses suggest a class effect benefit of 11–12% [1, 2], with this benefit restricted to a 14% improvement in participants with existing ASCVD [1]. SGLT2i appeared to have the largest benefit on the CV death component of MACE (HR 0.84 (0.75–0.94)) [2], and consistently reduce the risk of hHF (HR 0.65 to 0.73) [25–27] without increasing the risk of hypoglycemia. ACM was significantly reduced in only one SGLT2i CVOT to date [27], though there was a trend for benefit in all three completed trials [25, 26]. SGLT2i also consistently improved renal outcomes, with an estimated class benefit on worsening eGFR, ESRD, or renal death (HR 0.55 (0.48–0.64) [2]), regardless of ASCVD status and potentially also kidney disease status [1, 28]. In CREDENCE, canagliflozin reduced the relative risk of end-stage kidney disease, doubling of serum creatinine, or death from renal causes by 34%, and the relative risk of end-stage kidney disease by 32% in patients with type 2 diabetes [29].

These differences in outcomes with GLP-1 RA and SGLT2i are most likely tied to their mechanisms of action, over and above improvement of glycemia. GLP-1 RA enhance insulin secretion and inhibit glucagon secretion in a glucose-dependent manner, reduce appetite and gastric motility, increase heart rate, and exert natriuretic and vasodilatory effects in the kidney, contributing to weight loss and small blood pressure reductions [30, 31]. Effects on inflammation and endothelial function have also been observed in animals [31, 32], although clinical benefit of short-term exenatide treatment, compared to insulin glargine, on left ventricular function has not been observed [33]. SGLT2i act in an insulin-independent but glucose-dependent manner to increase urinary excretion of glucose, sodium, and water via osmotic diuresis [34–36]. These hemodynamic effects are subsequently hypothesized to reduce weight, blood pressure, and extracellular fluid via mechanisms distinct from and potentially complementary to renin-angiotensin-aldosterone system inhibitors [37]. SGLT2i also indirectly increase glucagon secretion and are hypothesized to alter inflammation, cardiac energetics, and metabolism [34–36]. These potentially complementary sets of mechanisms support the hypothesis that combination GLP-1 RA and SGLT2i treatment may results in further CV and renal benefit than observed with either class alone [5, 38, 39]. Results of the AWARD-10, SUSTAIN 9, and DURATION-8 trials and a subgroup analysis from CANVAS showed improvement in weight, triglycerides, and systolic blood pressure with different combinations of GLP-1 RA and SGLT2i, further increasing interest in this key question [8–10, 40, 41]; two additional trials are ongoing (LIRA-ADD2SGLT2i NCT02964247 and PIONEER4 NCT02863419). The results presented here support a potential added benefit for the combination of EQW and SGLT2i on ACM, at a minimum, an end-point on which both classes alone have shown some effect, and which may reflect a convergence of multiple mechanisms of action by these two drug classes. However, this analysis does not examine whether similar results would be observed with another GLP-1 RA.

The results in this analysis apply to a population with characteristics similar to the subpopulation in EXSCEL adding an SGLT2i as part of their usual glycemic care, as would be the case in a real-world analysis, and different from the populations typically found in a full randomized trial. More laboratory measurements were available here than are commonly found in registry data, allowing propensity matching based on these metrics and study visit. This approach avoids the time lag bias that can occur when comparing to participants initiating an anti-hyperglycemic medication that may be used as a different line of therapy [42]. Additionally, matching at comparable study visits avoids immortal time bias; subjects must remain in the trial for the same amount of time to be included, regardless of SGLT2i use [42]. Most participants in this analysis initiated EQW prior to an SGLT2i, in contrast to the GLP-1 RA + SGLT2i clinical trials performed to date [8–10, 40]. The propensity-matched cohorts also reflect regional patterns in SGLT2i use, with less history of CV disease but more albuminuria and a longer duration of diabetes than the overall EXSCEL trial population. Event rates were generally similar in these cohorts compared to the trial as a whole: 3.7 vs. 4.0 MACE per 100 participant-years, 2.0 vs. 2.3 ACM per 100 participant years, and 1.4 vs. 1.5 CV deaths per 100 participant-years in the EQW and placebo arms of EXSCEL, respectively [13]. These similarities in event rates are reassuring, though further study is required to assess the applicability of these results beyond the analyzed cohorts.

It is critical to consider the limitations of this analysis when interpreting the results. The *posthoc* nature of this analysis and the moderate sizes of the propensity-matched cohorts limit the statistical significance of our results; interpretation should focus on key, hypothesis-generating trends, and how they compare to existing knowledge in the field. While we carefully designed the propensity-matching procedure to balance patient characteristics, including metrics of disease state, medical history, laboratory measurements, and concomitant medications, we cannot exclude the possibility of bias due to unmeasured confounders [42–44]. Median follow-up time after SGLT2i initiation was under 2 years, restricting insight on CV outcomes and renal disease progression to this time-scale, which is relatively short for accrual of hard renal outcomes in this population with preserved renal function. We performed multiple analyses in two different cohorts, increasing the likelihood of type 1 error. To confirm the robustness of our results to matching variability within the study population, we re-matched and re-estimating hazard ratios; while one estimate for hHF was high relative to the resulting distribution (and thus likely a type 1 error), the estimates for MACE, ACM, and CV death were consistent with the overall distributions.

In EXSCEL, exact dates of SGLT2i initiation and cessation were not known [11]. As such, we used an “intent-to-treat”-like analysis, starting follow-up at the first known SGLT2i usage, as done previously [14]. In these cohorts, some patients added SGLT2i to their treatment plan while still taking study drug (exenatide or placebo), while others discontinued study drug (but remained in the trial) prior to SGLT2i initiation. This mix of participants, some using EQW and SGLT2i in sequence and others in parallel, is reflective of individual treatment decisions in clinical practice [45, 46]. In only subjects with overlapping EQW and SGLT2i use, we saw a similar benefit on mortality as in the primary analysis, though not on MACE. However, the limited size and resolution of this dataset constrains our ability to draw conclusions about sequential vs. parallel use of these drugs.

## Conclusions

This analysis provides the first insight, from a credible cohort with adjudicated CV events, into long-term cardiorenal outcomes in subjects using EQW and SGLT2i either in parallel or relatively close sequence. The results, supporting the hypothesis that this combination may provide additional benefit particularly on mortality, motivate additional, more comprehensive study of combination GLP-1 RA and SGLT2i in the future.

## Supplementary information

**Additional file 1: Table S1.** Total participant-years of SGLT2i exposure in the full EXSCEL trial by drug and trial arm. **Table S2.** Pre-match clinical characteristics of EXSCEL participants at trial baseline. **Table S3.** Detailed events, follow-up durations, and hazard ratios for exenatide + SGLT2i vs. placebo comparison in propensity-matched cohorts. **Table S4.** Detailed events, follow-up durations, and hazard ratios for exenatide + SGLT2i vs. exenatide comparison in propensity-matched cohorts. **Table S5.** Time-to-event exenatide QW + SGLT2i vs. placebo comparison in propensity-matched cohorts with overlapping SGLT2i and study drug use. **Table S6.** Time-to-event exenatide QW + SGLT2i vs. exenatide QW comparison in propensity-matched cohorts with overlapping SGLT2i and study drug use. **Table S7.** Time-to-event exenatide QW + SGLT2i vs. placebo comparison in propensity-matched cohorts censoring at open-label GLP-1 RA use. **Table S8.** Time-to-event exenatide QW + SGLT2i vs. exenatide QW comparison in propensity- matched cohorts censoring at open-label GLP-1 RA use. **Figure S1.** Participant flow chart. **Figure S2.** SGLT2i and study drug usage details. **Figure S3.** Balance of confounders before and after propensity matching. **Figure S4.** Propensity score distributions before and after matching. **Figure S5.** Kaplan–Meier curves for MACE, nonfatal MI, and nonfatal stroke. **Figure S6.** Kaplan–Meier curves for ACM and CV death. **Figure S7.** Kaplan–Meier curves for HHF and HHF + CV death. **Figure S8.** Kaplan–Meier curves for renal composites. **Figure S9.** Kaplan–Meier curves for serious hypoglycemia and amputation. **Figure S10.** Distributions of hazard ratio estimates when matching is repeated. **Figure S11.** Geometric mean (+/− standard error) eGFR slope before and after matching in the propensity-matched cohorts.

## Data Availability

Requests for data access and proposals for analyses of EXSCEL can be submitted to the EXSCEL executive committee using instructions found at: https://www.dtu.ox.ac.uk/exscel/.

## Abbreviations

ACM: all-cause mortality
aHR: adjusted hazard ratio
ASCVD: atherosclerotic cardiovascular disease
BMI: body mass index
CAD: coronary artery disease
CV: cardiovascular
CVD: cardiovascular disease
CVOT: cardiovascular outcomes trial
DPP-4i: dipeptidyl peptidase-4 inhibitors
eGFR: estimated glomerular filtration rate
ESRD: end-stage renal disease
EQW: exenatide once-weekly
EXSCEL: EXenatide Study of Cardiovascular Event Lowering
GLP1-RA: glucagon‑like peptide‑1 receptor agonists
HbA_1c_: glycated hemoglobin
HDL: high-density lipoproteins
hHF: hospitalization for heart failure
HR: hazard ratio
IQR: interquartile range
LDL: low-density lipoproteins
MACE: major adverse cardiovascular events
MDRD: modification of diet in renal disease
MMRM: mixed-model repeated measures
PAD: peripheral artery disease
QW: once weekly
RAASi: renin-angiotensin-aldosterone system inhibitors
SD: standard deviation
SGLT2i: sodium-glucose co-transporter-2 inhibitors
TZD: thiazolidinediones
UACR: urinary albumin-to-creatine ratio

## References

[1] Zelniker TA, Wiviott SD, Raz I, Im K, Goodrich EL, Bonaca MP (2019). SGLT2 inhibitors for primary and secondary prevention of cardiovascular and renal outcomes in type 2 diabetes: a systematic review and meta-analysis of cardiovascular outcome trials. Lancet.

[2] Zelniker Thomas A, Wiviott Stephen D, Raz I, Im K, Goodrich Erica L, Furtado Remo HM (2019). Comparison of the effects of glucagon-like peptide receptor agonists and sodium-glucose co-transporter 2 inhibitors for prevention of major adverse cardiovascular and renal outcomes in type 2 diabetes mellitus: a systematic review and meta-analysis of cardiovascular outcomes trials. Circulation.

[3] Hussein H, Zaccardi F, Khunti K, Seidu S, Davies MJ, Gray LJ (2019). Cardiovascular efficacy and safety of sodium-glucose co-transporter-2 inhibitors and glucagon-like peptide-1 receptor agonists: a systematic review and network meta-analysis. Diabetic Med.

[4] Giugliano D, Maiorino MI, Bellastella G, Longo M, Chiodini P, Esposito K (2019). GLP-1 receptor agonists for prevention of cardiorenal outcomes in type 2 diabetes: an updated meta-analysis including the REWIND and PIONEER 6 trials. Diabetes Obes Metab.

[5] DeFronzo RA (2017). Combination therapy with GLP-1 receptor agonist and SGLT2 inhibitor. Diabetes Obes Metab.

[6] Goncalves E, Bell DSH (2018). Combination treatment of SGLT2 inhibitors and GLP-1 receptor agonists: symbiotic effects on metabolism and cardiorenal risk. Diabetes Ther.

[7] Frías JP, Guja C, Hardy E, Ahmed A, Dong F, Öhman P (2016). Exenatide once weekly plus dapagliflozin once daily versus exenatide or dapagliflozin alone in patients with type 2 diabetes inadequately controlled with metformin monotherapy (DURATION-8): a 28 week, multicentre, double-blind, phase 3, randomised controlled trial. Lancet Diabetes Endocrinol.

[8] Ludvik B, Frías JP, Tinahones FJ, Wainstein J, Jiang H, Robertson KE (2018). Dulaglutide as add-on therapy to SGLT2 inhibitors in patients with inadequately controlled type 2 diabetes (AWARD-10): a 24-week, randomised, double-blind, placebo-controlled trial. Lancet Diabetes Endocrinol.

[9] Zinman B, Bhosekar V, Busch R, Holst I, Ludvik B, Thielke D (2019). Semaglutide once weekly as add-on to SGLT-2 inhibitor therapy in type 2 diabetes (SUSTAIN 9): a randomised, placebo-controlled trial. Lancet Diabetes Endocrinol.

[10] Hardy E, Öhman P, Jabbour S, Guja C, Frias J, Bhattacharya S, editors. DURATION-8 randomized controlled trial 104-week results: efficacy and safety of once-weekly exenatide (ExQW) plus once-daily dapagliflozin (DAPA) vs ExQW or DAPA alone. European Association for the Study of Diabetes; 2018; Berlin, Germany.

[11] Holman RR, Bethel MA, George J, Sourij H, Doran Z, Keenan J (2016). Rationale and design of the EXenatide Study of Cardiovascular Event Lowering (EXSCEL) trial. Am Heart J.

[12] Mentz RJ, Bethel MA, Gustavson S, Thompson VP, Pagidipati NJ, Buse JB (2017). Baseline characteristics of patients enrolled in the Exenatide Study of Cardiovascular Event Lowering (EXSCEL). Am Heart J.

[13] Holman RR, Bethel MA, Mentz RJ, Thompson VP, Lokhnygina Y, Buse JB (2017). Effects of once-weekly exenatide on cardiovascular outcomes in type 2 diabetes. N Engl J Med.

[14] Clegg LE, Heerspink HJL, Penland RC, Tang W, Boulton DW, Bachina S (2019). Reduction of cardiovascular risk and improved estimated glomerular filtration rate by SGLT2 inhibitors, including dapagliflozin, is consistent across the class: an analysis of the placebo arm of EXSCEL. J Diabetes Care.

[15] Stuart DEHaKIaGKaEA (2011). MatchIt: nonparametric preprocessing for parametric casual inference. J Stat Softw.

[16] Funk MJ, Westreich D, Wiesen C, Stürmer T, Brookhart MA, Davidian M (2011). Doubly robust estimation of causal effects. Am J Epidemiol.

[17] Raab GM, Day S, Sales J (2000). How to select covariates to include in the analysis of a clinical trial. Control Clin Trials.

[18] Levey AS, Coresh J, Greene T (2006). USing standardized serum creatinine values in the modification of diet in renal disease study equation for estimating glomerular filtration rate. Ann Intern Med.

[19] Team RC (2017). R: A language and environment for statistical computing.

[20] Bethel MA, Patel RA, Merrill P, Lokhnygina Y, Buse JB, Mentz RJ (2018). Cardiovascular outcomes with glucagon-like peptide-1 receptor agonists in patients with type 2 diabetes: a meta-analysis. Lancet Diabetes Endocrinol.

[21] Gerstein HC, Colhoun HM, Dagenais GR, Diaz R, Lakshmanan M, Pais P (2019). Dulaglutide and cardiovascular outcomes in type 2 diabetes (REWIND): a double-blind, randomised placebo-controlled trial. Lancet.

[22] Mann JFE, Ørsted DD, Brown-Frandsen K, Marso SP, Poulter NR, Rasmussen S (2017). Liraglutide and renal outcomes in type 2 diabetes. N Engl J Med.

[23] Gerstein HC, Colhoun HM, Dagenais GR, Diaz R, Lakshmanan M, Pais P (2019). Dulaglutide and renal outcomes in type 2 diabetes: an exploratory analysis of the REWIND randomised, placebo-controlled trial. Lancet.

[24] Marso SP, Bain SC, Consoli A, Eliaschewitz FG, Jódar E, Leiter LA (2016). Semaglutide and cardiovascular outcomes in patients with type 2 diabetes. N Engl J Med.

[25] Wiviott SD, Raz I, Bonaca MP, Mosenzon O, Kato ET, Cahn A (2019). Dapagliflozin and cardiovascular outcomes in type 2 diabetes. N Engl J Med.

[26] Neal B, Perkovic V, Mahaffey KW, de Zeeuw D, Fulcher G, Erondu N (2017). Canagliflozin and cardiovascular and renal events in type 2 diabetes. N Engl J Med.

[27] Zinman B, Wanner C, Lachin JM, Fitchett D, Bluhmki E, Hantel S (2015). Empagliflozin, cardiovascular outcomes, and mortality in type 2 diabetes. N Engl J Med.

[28] Lo C, Toyama T, Wang Y, Lin J, Hirakawa Y, Jun M (2018). Insulin and glucose-lowering agents for treating people with diabetes and chronic kidney disease. Cochrane Database Syst Rev.

[29] Perkovic V, Jardine MJ, Neal B, Bompoint S, Heerspink HJL, Charytan DM (2019). Canagliflozin and renal outcomes in type 2 diabetes and nephropathy. N Engl J Med.

[30] Rizzo M, Nikolic D, Patti AM, Mannina C, Montalto G, McAdams BS (2018). GLP-1 receptor agonists and reduction of cardiometabolic risk: potential underlying mechanisms. Biochim Biophys Acta.

[31] Drucker DJ (2018). The ascending GLP-1 road from clinical safety to reduction of cardiovascular complications. Diabetes.

[32] Muskiet Marcel H. A., Smits Mark M., Morsink Linde M., Diamant Michaela (2013). The gut–renal axis: do incretin-based agents confer renoprotection in diabetes?. Nature Reviews Nephrology.

[33] Chen WJY, Diamant M, de Boer K, Harms HJ, Robbers LFHJ, van Rossum AC (2017). Effects of exenatide on cardiac function, perfusion, and energetics in type 2 diabetic patients with cardiomyopathy: a randomized controlled trial against insulin glargine. Cardiovasc Diabetol.

[34] Hallow KM, Helmlinger G, Greasley PJ, McMurray JJ, Boulton DW (2018). Why do SGLT2 inhibitors reduce heart failure hospitalization? A differential volume regulation hypothesis. Diabetes Obes Metab.

[35] Fioretto P, Zambon A, Rossato M, Busetto L, Vettor R (2016). SGLT2 inhibitors and the diabetic kidney. Diabetes Care.

[36] Staels B (2017). Cardiovascular protection by sodium glucose cotransporter 2 Inhibitors: potential mechanisms. Am J Med.

[37] Zou H, Zhou B, Xu G (2017). SGLT2 inhibitors: a novel choice for the combination therapy in diabetic kidney disease. Cardiovasc Diabetol.

[38] Goncalves E, Bell DSH (2018). Combination treatment of SGLT2 inhibitors and GLP-1 receptor agonists: symbiotic effects on metabolism and cardiorenal risk. Diabetes Ther.

[39] Heerspink HJL, Perkins BA, Fitchett DH, Husain M, Cherney DZI (2016). Sodium glucose cotransporter 2 inhibitors in the treatment of diabetes mellitus: cardiovascular and kidney effects, potential mechanisms, and clinical applications. Circulation.

[40] Fulcher G, Matthews DR, Perkovic V, de Zeeuw D, Mahaffey KW, Mathieu C (2016). Efficacy and safety of canagliflozin when used in conjunction with incretin-mimetic therapy in patients with type 2 diabetes. Diabetes Obes Metab.

[41] Jabbour SA, Frías JP, Guja C, Hardy E, Ahmed A, Öhman P (2018). Effects of exenatide once weekly plus dapagliflozin, exenatide once weekly, or dapagliflozin, added to metformin monotherapy, on body weight, systolic blood pressure, and triglycerides in patients with type 2 diabetes in the DURATION-8 study. Diabetes Obes Metab.

[42] Suissa S (2018). Lower risk of death with SGLT2 inhibitors in observational studies: real or bias?. Diabetes Care.

[43] Austin PC (2008). A critical appraisal of propensity-score matching in the medical literature between 1996 and 2003. Stat Med.

[44] Austin PC, Grootendorst P, Anderson GM (2007). A comparison of the ability of different propensity score models to balance measured variables between treated and untreated subjects: a Monte Carlo study. Stat Med.

[45] Goncalves E, Bell DSH (2017). Glucagon-like peptide-1 receptor agonists and sodium-glucose co-transporter-2 inhibitors: sequential or simultaneous start?. Diabetes Obes Metab.

[46] Saroka RM, Kane MP, Busch RS, Watsky J, Hamilton RA (2015). SGLT-2 inhibitor therapy added TO GLP-1 agonist therapy in the management of T2DM. Endocrine Pract.

