# Supplementary material for: Effects of exenatide and open-label SGLT2 inhibitor treatment, given in parallel or sequentially, on mortality and cardiovascular and renal outcomes in type 2 diabetes: insights from the EXSCEL trial
Source: Cardiovasc Diabetol. 2019 Oct 22;18:138. doi: 10.1186/s12933-019-0942-x (PMC6805385; doi:10.1186/s12933-019-0942-x)
Supplement: Supplementary file 1 — Additional file 1: Table S1. Total participant-years of SGLT2i exposure in the full EXSCEL trial by drug and trial arm. Table S2. Pre-match clinical characteristics of EXSCEL participants at trial baseline. Table S3. Detailed events, follow-up durations, and hazard ratios for exenatide + SGLT2i vs. placebo comparison in propensity-matched cohorts. Table S4. Detailed events, follow-up durations, and hazard ratios for exenatide + SGLT2i vs. exenatide comparison in propensity-matched cohorts. Table S5. Time-to-event exenatide QW + SGLT2i vs. placebo comparison in propensity-matched cohorts with overlapping SGLT2i and study drug use. Table S6. Time-to-event exenatide QW + SGLT2i vs. exenatide QW comparison in propensity-matched cohorts with overlapping SGLT2i and study drug use. Table S7. Time-to-event exenatide QW + SGLT2i vs. placebo comparison in propensity-matched cohorts censoring at open-label GLP-1 RA use. Table S8. Time-to-event exenatide QW + SGLT2i vs. exenatide QW comparison in propensity- matched cohorts censoring at open-label GLP-1 RA use. Figure S1. Participant flow chart. Figure S2. SGLT2i and study drug usage details. Figure S3. Balance of confounders before and after propensity matching. Figure S4. Propensity score distributions before and after matching. Figure S5. Kaplan–Meier curves for MACE, nonfatal MI, and nonfatal stroke. Figure S6. Kaplan–Meier curves for ACM and CV death. Figure S7. Kaplan–Meier curves for HHF and HHF + CV death. Figure S8. Kaplan–Meier curves for renal composites. Figure S9. Kaplan–Meier curves for serious hypoglycemia and amputation. Figure S10. Distributions of hazard ratio estimates when matching is repeated. Figure S11. Geometric mean (+/− standard error) eGFR slope before and after matching in the propensity-matched cohorts. [file 12933_2019_942_MOESM1_ESM.docx]

**Supplemental Information**

**Supplemental Tables**

**Table S1.** Total participant-years of SGLT2i exposure in the full EXSCEL trial by drug and trial arm.

**Table S2.** Pre-match clinical characteristics of EXSCEL participants at trial baseline.

**Table S3.** Detailed events, follow-up durations, and hazard ratios for exenatide + SGLT2i vs. placebo comparison in propensity-matched cohorts.

**Table S4.** Detailed events, follow-up durations, and hazard ratios for exenatide + SGLT2i vs. exenatide comparison in propensity-matched cohorts.

**Table S5.** Time-to-event exenatide QW + SGLT2i vs. placebo comparison in propensity-matched cohorts with overlapping SGLT2i and study drug use.

**Table S6.** Time-to-event exenatide QW + SGLT2i vs. exenatide QW comparison in propensity-matched cohorts with overlapping SGLT2i and study drug use.

**Table S7.** Time-to-event exenatide QW + SGLT2i vs. placebo comparison in propensity-matched cohorts censoring at open-label GLP-1 RA use.

**Table S8.** Time-to-event exenatide QW + SGLT2i vs. exenatide QW comparison in propensity- matched cohorts censoring at open-label GLP-1 RA use.

**Supplemental Figures**

**Figure S1.** Participant flow chart.

**Figure S2.** SGLT2i and study drug usage details.

**Figure S3.** Balance of confounders before and after propensity matching.

**Figure S4.** Propensity score distributions before and after matching.

**Figure S5.** Kaplan-Meier curves for MACE, nonfatal MI, and nonfatal stroke.

**Figure S6.** Kaplan-Meier curves for ACM and CV death.

**Figure S7.** Kaplan-Meier curves for HHF and HHF + CV death.

**Figure S8.** Kaplan-Meier curves for renal composites.

**Figure S9.** Kaplan-Meier curves for serious hypoglycemia and amputation.

**Figure S10.** Distributions of hazard ratio estimates when matching is repeated.

**Figure S11.** Geometric mean (+/- standard error) eGFR slope before and after matching in the propensity-matched cohorts.

**Supplemental Tables**

**Table S1.** Total participant-years of SGLT2i exposure in the full EXSCEL trial by drug and trial arm.

|  | **Placebo** | **Exenatide QW** |
| --- | --- | --- |
| **All SGLT2i** | 732 | 614 |
| **dapagliflozin only** | 314 | 271 |
| **canagliflozin only** | 211 | 178 |
| **empagliflozin only** | 101 | 72 |
| **Multiple*** | 106 | 93 |

Calculated as time from first known SGLT2i use to last known SGLT2i use, regardless of gaps. SGLT2i, sodium-glucose co-transporter-2 inhibitor; QW, once weekly. ^*^Multiple includes participants who took more than one SGLT2i over the course of the trial.

**Table S2.** Pre-match clinical characteristics of EXSCEL participants at trial baseline.

|  | **Placebo,  No SGLT2i** | **Exenatide QW,  No SGLT2i** | **Exenatide QW + SGLT2i** |
| --- | --- | --- | --- |
| **Participants, n** | 6610 | 6,711 | 645 |
| **Sex, male** | 4056 (61%) | 4,122 (61%) | 440 (68%) |
| **Age, years** | 62 (9) | 62 (9) | 60 (9) |
| **Race** |  |  |  |
| **White** | 4939 (75%) | 5,011 (75%) | 543 (84%) |
| **Black** | 414 (6.3%) | 419 (6.2%) | 23 (3.6%) |
| **Asian** | 672 (10%) | 667 (9.9%) | 58 (9.0%) |
| **Other/Unknown** | 585 (8.9%) | 614 (9.1%) | 21 (3.3%) |
| **Region** |  |  |  |
| **North America** | 1591 (24%) | 1,589 (24%) | 245 (38%) |
| **Latin America** | 1312 (20%) | 1,334 (20%) | 30 (4.7%) |
| **Asia Pacific** | 699 (11%) | 711 (11%) | 58 (9.0%) |
| **Western Europe** | 1148 (17%) | 1,161 (17) | 113 (33%) |
| **Eastern Europe** | 1860 (28%) | 1,916 (29%) | 99 (15%) |
| **Ethnicity, Hispanic** | 1451 (22%) | 1,465 (22%) | 41 (6.4%) |
| **Duration of diabetes, years** | 13 (8) | 13 (8) | 13 (8) |
| **History of CVD (CAD, PAD, or stroke)** | 4887 (74%) | 4,993 (74%) | 401 (62%) |
| **History of heart failure** | 1131 (17%) | 1,102 (16%) | 59 (9.1%) |
| **History of retinopathy** | 1137 (17%) | 1,171 (17%) | 99 (15%) |
| **History of albuminuria** | 1012 (15%) | 1,059 (16%) | 146 (23%) |
| **Microalbuminuria** | 777 (12%) | 854 (13%) | 127 (20%) |
| **Macroalbuminuria** | 235 (3.6%) | 205 (3.1%) | 19 (2.9%) |
| **Systolic blood pressure, mmHg** | 135.6 (17.0) | 135.5 (17.0) | 134.5 (15.1) |
| **Diastolic blood pressure, mmHg** | 77.9 (10.2) | 78.3 (10.4) | 78.5 (9.9) |
| **BMI, kg/m^2^** | 32.5 (6.5) | 32.5 (6.3) | 34.5 (6.4) |
| **HbA_1c_, %** | 8.1 (1.0) | 8.1 (1.0) | 8.3 (0.9) |
| **mmol/mol** | 65 | 65 | 67 |
| **Cholesterol, mmol/L** | 4.6 (3.1) | 4.5 (1.3) | 4.3 (1.1) |
| **LDL, mmol/L** | 2.5 (1.9) | 2.5 (1.0) | 2.3 (0.9) |
| **HDL, mmol/L** | 1.1 (0.3) | 1.2 (0.9) | 1.1 (0.3) |
| **UACR (median, IQR), g/mol** | 1.7 [0.5,6.3] | 1.3 [0.5,4.7] | 1.5 [0.4,5.5] |
| **Hemoglobin, g/L** | 141.4 (75.9) | 138.2 (15.6) | 140.4 (15.5) |
| **eGFR, mL/min/1.73 m^2^** | 75.9 (24.2) | 76.4 (23.4) | 84.5 (22.5) |
| **eGFR<60 mL/min/1.73 m^2^** | 1700 (26%) | 1,646 (25%) | 83 (13%) |
| **eGFR<45 mL/min/1.73 m^2^** | 516 (7.8%) | 465 (6.9%) | 7 (1.1%) |
| **Smoking** |  |  |  |
| **Never** | 766 (12%) | 772 (12%) | 92 (14%) |
| **Past** | 2544 (38%) | 2,640 (39%) | 262 (41%) |
| **Current** | 3296 (50%) | 3,296 (49%) | 291 (45%) |
| **Classes of diabetes medications (n)^*^** | 1.3 (0.8) | 1.3 (0.8) | 1.7 (0.9) |
| **RAASi** | 5153 (78%) | 5,217 (78%) | 518 (80%) |
| **Other antihypertensives** | 3831 (58%) | 3,872 (58%) | 371 (58%) |
| **Statin** | 4759 (72%) | 4,949 (74%) | 502 (78%) |
| **Diuretic** | 2893 (44%) | 2,942 (44%) | 268 (42%) |
| **Insulin** | 3079 (47%) | 3,080 (46%) | 308 (48%) |
| **Metformin** | 4993 (76%) | 5,049 (75%) | 555 (86%) |
| **TZD** | 236 (3.6%) | 241 (3.6%) | 51 (7.9%) |
| **DPP-4i** | 863 (13%) | 913 (14%) | 204 (32%) |
| **Sulfonylureas** | 2410 (36%) | 2,442 (36%) | 250 (39%) |

Continuous metrics are reported as mean (SD). Categorical metrics are reported as n (%). ^*^Classes of anti-hyperglycemic agents included: biguanides, sulfonylureas, meglitinides, DPP-4i, and TZD. Insulin, SGLT2i, and GLP1-RA (excluded by study protocol) are not included**.**

BMI, body mass index; CAD, coronary artery disease; CVD, cardiovascular disease; DPP-4i, dipeptidyl peptidase-4 inhibitors; eGFR, estimated glomerular filtration rate; GLP1-RA, glucagon‑like peptide‑1 receptor agonists; HbA_1c_, glycated hemoglobin; HDL, high-density lipoproteins; LDL, low-density lipoproteins; PAD, peripheral artery disease; RAASi, renin-angiotensin-aldosterone system inhibitors; SD, standard deviation; SGLT2i, sodium-glucose co-transporter-2 inhibitors; TZD, thiazolidinediones; UACR, urinary albumin-to-creatine ratio; QW, once weekly.

**Table S3.** Detailed events, follow-up durations, and hazard ratios for exenatide QW + SGLT2i vs. placebo comparison in propensity-matched cohorts.

| **Time-to-first adjudicated event** | **Propensity-matched cohort** | **n** | **Events** | **Participant-years of follow-up** | **Incidence rate (events/100 participant-years)** | **Unadjusted hazard ratio  (95% CI)** | **Adjusted hazard ratio^†^  (95% CI)** | **Nominal p-values (adjusted)** |
| --- | --- | --- | --- | --- | --- | --- | --- | --- |
| **MACE** | Placebo, No SGLT2i | 572 | 36 | 748 | 4.81 | 0.67 | 0.68 | 0.16 |
|  | Exenatide + SGLT2i | 572 | 21 | 639 | 3.29 | (0.39-1.15) | (0.39-1.17) |  |
| **ACM** | Placebo, No SGLT2i | 572 | 23 | 848 | 2.71 | 0.35 | 0.38 | 0.03 |
|  | Exenatide + SGLT2i | 572 | 7 | 703 | 1.00 | (0.15-0.83) | (0.16-0.90) |  |
| **hHF** | Placebo, No SGLT2i | 572 | 5 | 806 | 0.62 | 1.11 | 0.98 | 0.98 |
|  | Exenatide + SGLT2i | 572 | 5 | 682 | 0.73 | (0.32-3.88) | (0.28-3.45) |  |
| **CV Death** | Placebo, No SGLT2i | 572 | 14 | 848 | 1.65 | 0.16 | 0.17 | 0.02 |
|  | Exenatide + SGLT2i | 572 | 2 | 703 | 0.28 | (0.04-0.70) | (0.04-0.77) |  |
| **Nonfatal MI** | Placebo, No SGLT2i | 572 | 21 | 772 | 2.72 | 0.83 | 0.88 | 0.71 |
|  | Exenatide + SGLT2i | 572 | 15 | 647 | 2.32 | (0.43-1.63) | (0.44-1.74) |  |
| **Nonfatal** | Placebo, No SGLT2i | 572 | 7 | 792 | 0.88 | 0.92 | 1.10 | 0.88 |
| **Stroke** | Exenatide + SGLT2i | 572 | 5 | 683 | 0.73 | (0.28-3.07) | (0.32-3.81) |  |
| **hHF +** | Placebo, No SGLT2i | 572 | 19 | 843 | 2.25 | 0.41 | 0.41 | 0.045 |
| **CV Death** | Exenatide + SGLT2i | 572 | 7 | 700 | 1.00 | (0.17-0.99) | (0.17-0.98) |  |
| **Renal_1*** | Placebo, No SGLT2i | 362 | 6 | 616 | 0.97 | 0.32 | 0.30 | 0.15 |
|  | Exenatide + SGLT2i | 437 | 2 | 585 | 0.34 | (0.06-1.59) | (0.06-1.52) |  |
| **Renal_2**** | Placebo, No SGLT2i | 342 | 12 | 581 | 2.07 | 0.43 | 0.42 | 0.11 |
|  | Exenatide + SGLT2i | 417 | 5 | 556 | 0.90 | (0.15-1.22) | (0.15-1.22) |  |
| **Serious** | Placebo, No SGLT2i | 572 | 11 | 871 | 1.25 | 0.69 | 0.67 | 0.42 |
| **hypoglycemia** | Exenatide + SGLT2i | 572 | 7 | 746 | 0.94 | (0.26-1.79) | (0.26-1.76) |  |
| **Amputation** | Placebo, No SGLT2i | 572 | 2 | 866 | 0.23 | 1.54 | 1.18 | 0.86 |
|  | Exenatide + SGLT2i | 572 | 3 | 747 | 0.40 | (0.26-9.25) | (0.18-7.65) |  |

*Renal_1 evaluated in participants with at least two eGFR measurements after the time of matching. **Renal_2 evaluated in participants included in Renal_1 without macroalbuminuria at the time of matching.

MACE, major adverse cardiovascular events; ACM, all-cause mortality; hHF, hospitalization for heart failure; SGLT2i, sodium-glucose co-transporter-2 inhibitor. **^†^**Adjustment for: duration of diabetes, age, sex, history of CVD, prior heart failure, prior microalbuminuria, prior macroalbuminuria, baseline eGFR, and baseline HbA_1c_.

**Table S4.** Detailed events, follow-up durations, and hazard ratios for exenatide QW + SGLT2i vs. exenatide QW comparison in propensity-matched cohorts.

| **Time-to-first adjudicated event** | **Propensity-matched cohort** | **n** | **Events** | **Participant-years of follow-up** | **Incidence rate (events/100 participant-years)** | **Unadjusted hazard ratio  (95% CI)** | **Adjusted hazard ratio^†^  (95% CI)** | **Nominal p-values (adjusted)** |
| --- | --- | --- | --- | --- | --- | --- | --- | --- |
| **MACE** | Exenatide, No SGLT2i | 575 | 33 | 753 | 4.38 | 0.79 | 0.85 | 0.56 |
|  | Exenatide + SGLT2i | 575 | 21 | 643 | 3.27 | (0.45-1.39) | (0.48-1.49) |  |
| **ACM** | Exenatide, No SGLT2i | 575 | 25 | 846 | 2.96 | 0.36 | 0.41 | 0.04 |
|  | Exenatide + SGLT2i | 575 | 7 | 707 | 0.99 | (0.15-0.84) | (0.17-0.95) |  |
| **hHF** | Exenatide, No SGLT2i | 575 | 12 | 798 | 1.50 | 0.46 | 0.51 | 0.22 |
|  | Exenatide + SGLT2i | 575 | 5 | 684 | 0.73 | (0.16-1.30) | (0.18-1.48) |  |
| **CV Death** | Exenatide, No SGLT2i | 575 | 15 | 846 | 1.77 | 0.19 | 0.21 | 0.04 |
|  | Exenatide + SGLT2i | 575 | 2 | 707 | 0.28 | (0.04-0.83) | (0.05-0.93) |  |
| **Nonfatal MI** | Exenatide, No SGLT2i | 575 | 13 | 768 | 1.69 | 1.23 | 1.31 | 0.48 |
|  | Exenatide + SGLT2i | 575 | 15 | 652 | 2.30 | (0.58-2.59) | (0.62-2.78) |  |
| **Nonfatal** | Exenatide, No SGLT2i | 575 | 10 | 799 | 1.25 | 0.66 | 0.68 | 0.49 |
| **Stroke** | Exenatide + SGLT2i | 575 | 5 | 685 | 0.73 | (0.22-1.98) | (0.22-2.05) |  |
| **hHF +** | Exenatide, No SGLT2i | 575 | 24 | 838 | 2.86 | 0.37 | 0.41 | 0.04 |
| **CV Death** | Exenatide + SGLT2i | 575 | 7 | 704 | 0.99 | (0.16-0.87) | (0.17-0.95) |  |
| **Renal_1*** | Exenatide, No SGLT2i | 397 | 10 | 657 | 1.52 | 0.21 | 0.22 | 0.051 |
|  | Exenatide + SGLT2i | 437 | 2 | 585 | 0.34 | (0.05-0.97) | (0.05-1.00) |  |
| **Renal_2**** | Exenatide, No SGLT2i | 373 | 15 | 614 | 2.44 | 0.35 | 0.39 | 0.07 |
|  | Exenatide + SGLT2i | 417 | 5 | 556 | 0.90 | (0.13-0.98) | (0.14-1.09) |  |
| **Serious** | Exenatide, No SGLT2i | 575 | 11 | 880 | 1.25 | 0.71 | 0.77 | 0.59 |
| **hypoglycemia** | Exenatide + SGLT2i | 575 | 7 | 751 | 0.93 | (0.27-1.86) | (0.30-1.99) |  |
| **Amputation** | Exenatide, No SGLT2i | 575 | 2 | 860 | 0.23 | 1.56 | 1.75 | 0.56 |
|  | Exenatide + SGLT2i | 575 | 3 | 750 | 0.40 | (0.26-9.38) | (0.27-11.39) |  |

*Renal_1 evaluated in participants with at least two eGFR measurements after the time of matching. **Renal_2 evaluated in participants included in Renal_1 without macroalbuminuria at the time of matching.

MACE, major adverse cardiovascular events; ACM, all-cause mortality; hHF, hospitalization for heart failure; SGLT2i, sodium-glucose co-transporter-2 inhibitor. **^†^**Adjustment for: duration of diabetes, age, sex, history of CVD, prior heart failure, prior microalbuminuria, prior macroalbuminuria, baseline eGFR, and baseline HbA_1c_.

**Table S5.** Time-to-event exenatide QW + SGLT2i vs. placebo comparison in propensity-matched cohorts with overlapping SGLT2i and study drug use.

| **Time-to-first adjudicated event** | **Propensity-matched cohort** | **n** | **Events** | **Participant-years of follow-up** | **Incidence rate (events/100 participant-years)** | **Unadjusted hazard ratio  (95% CI)** | **Adjusted hazard ratio^†^  (95% CI)** | **Nominal p-values (adjusted)** |
| --- | --- | --- | --- | --- | --- | --- | --- | --- |
| **MACE** | Placebo, No SGLT2i | 340 | 19 | 504 | 3.77 | 1.29 | 1.38 | 0.37 |
|  | Exenatide + SGLT2i | 340 | 18 | 430 | 4.19 | (0.65-2.53) | (0.68-2.81) |  |
| **ACM** | Placebo, No SGLT2i | 340 | 19 | 564 | 3.37 | 0.47 | 0.47 | 0.11 |
|  | Exenatide + SGLT2i | 340 | 7 | 454 | 1.54 | (0.19-1.15) | (0.19-1.18) |  |
| **CV Death** | Placebo, No SGLT2i | 340 | 11 | 564 | 1.95 | 0.42 | 0.45 | 0.24 |
|  | Exenatide + SGLT2i | 340 | 3 | 454 | 0.66 | (0.11-1.59) | (0.11-1.74) |  |

p-value in a chi-squared omnibus test for balance was 0.971. MACE, major adverse cardiovascular events; SGLT2i, sodium-glucose co-transporter-2 inhibitor. **^†^**Adjustment for: duration of diabetes, age, sex, history of CVD, prior heart failure, prior microalbuminuria, prior macroalbuminuria, baseline eGFR, and baseline HbA_1c_.

**Table S6.** Time-to-event exenatide QW + SGLT2i vs. exenatide QW comparison in propensity-matched cohorts with overlapping SGLT2i and study drug use.

| **Time-to-first adjudicated event** | **Propensity-matched cohort** | **n** | **Events** | **Participant-years of follow-up** | **Incidence rate (events/100 participant-years)** | **Unadjusted hazard ratio  (95% CI)** | **Adjusted hazard ratio^†^  (95% CI)** | **Nominal p-values (adjusted)** |
| --- | --- | --- | --- | --- | --- | --- | --- | --- |
| **MACE** | Exenatide, No SGLT2i | 336 | 19 | 528 | 3.60 | 1.22 | 1.24 | 0.53 |
|  | Exenatide + SGLT2i | 336 | 18 | 426 | 4.23 | (0.62-2.37) | (0.63-2.44) |  |
| **ACM** | Exenatide, No SGLT2i | 336 | 15 | 587 | 2.56 | 0.58 | 0.56 | 0.21 |
|  | Exenatide + SGLT2i | 336 | 7 | 451 | 1.55 | (0.23-1.44) | (0.22-1.40) |  |
| **CV Death** | Exenatide, No SGLT2i | 336 | 12 | 587 | 2.04 | 0.30 | 0.28 | 0.05 |
|  | Exenatide + SGLT2i | 336 | 3 | 451 | 0.67 | (0.08-1.10) | (0.08-1.02) |  |

p-value in a chi-squared omnibus test for balance was 0.996. MACE, major adverse cardiovascular events; SGLT2i, sodium-glucose co-transporter-2 inhibitor. **^†^**Adjustment for: duration of diabetes, age, sex, history of CVD, prior heart failure, prior microalbuminuria, prior macroalbuminuria, baseline eGFR, and baseline HbA_1c_.

**Table S7.** Time-to-event exenatide QW + SGLT2i vs. placebo comparison in propensity-matched cohorts censoring at open-label GLP-1 RA use.

| **Time-to-first adjudicated event** | **Propensity-matched cohort** | **n** | **Events** | **Participant-years of follow-up** | **Incidence rate (events/100 participant-years)** | **Unadjusted hazard ratio  (95% CI)** | **Adjusted hazard ratio^†^  (95% CI)** | **Nominal p-values (adjusted)** |
| --- | --- | --- | --- | --- | --- | --- | --- | --- |
| **MACE** | Placebo, No SGLT2i | 572 | 36 | 717 | 5.02 | 0.70 | 0.71 | 0.22 |
|  | Exenatide + SGLT2i | 572 | 21 | 589 | 3.57 | (0.40-1.21) | (0.41-1.23) |  |
| **ACM** | Placebo, No SGLT2i | 572 | 23 | 811 | 2.84 | 0.37 | 0.40 | 0.04 |
|  | Exenatide + SGLT2i | 572 | 7 | 642 | 1.09 | (0.16-0.87) | (0.17-0.96) |  |
| **CV Death** | Placebo, No SGLT2i | 572 | 14 | 811 | 1.73 | 0.16 | 0.18 | 0.02 |
|  | Exenatide + SGLT2i | 572 | 2 | 642 | 0.31 | (0.04-0.73) | (0.04-0.80) |  |
| **Serious Hypoglycemia** | Placebo, No SGLT2i | 572 | 11 | 834 | 1.32 | 0.62 | 0.60 | 0.31 |
|  | Exenatide + SGLT2i | 572 | 6 | 686 | 0.87 | (0.23-1.70) | (0.22-1.64) |  |

Participants starting open-label GLP-1 RA before matching in placebo cohort (n=17) and combination cohort (n=26) were censored at time zero. Participants starting open-label GLP-1 RA at or after matching in placebo cohort (n=16) and combination cohort (n=26) were censored upon initiation of GLP-1 RA (including exenatide) other than study drug.

MACE, major adverse cardiovascular events; SGLT2i, sodium-glucose co-transporter-2 inhibitor. **^†^**Adjustment for: duration of diabetes, age, sex, history of CVD, prior heart failure, prior microalbuminuria, prior macroalbuminuria, baseline eGFR, and baseline HbA_1c_.

**Table S8.** Time-to-event exenatide QW + SGLT2i vs. exenatide comparison in propensity-matched cohorts censoring at open-label GLP-1 RA use.

| **Time-to-first adjudicated event** | **Propensity-matched cohort** | **n** | **Events** | **Participant-years of follow-up** | **Incidence rate (events/100 participant-years)** | **Unadjusted hazard ratio  (95% CI)** | **Adjusted hazard ratio^†^  (95% CI)** | **Nominal p-values (adjusted)** |
| --- | --- | --- | --- | --- | --- | --- | --- | --- |
| **MACE** | Exenatide, No SGLT2i | 575 | 33 | 727 | 4.54 | 0.84 | 0.88 | 0.65 |
|  | Exenatide + SGLT2i | 575 | 21 | 591 | 3.55 | (0.48-1.48) | (0.50-1.55) |  |
| **ACM** | Exenatide, No SGLT2i | 575 | 25 | 817 | 3.06 | 0.38 | 0.41 | 0.04 |
|  | Exenatide + SGLT2i | 575 | 7 | 647 | 1.08 | (0.16-0.90) | (0.18-0.97) |  |
| **CV Death** | Exenatide, No SGLT2i | 575 | 15 | 817 | 1.84 | 0.20 | 0.21 | 0.04 |
|  | Exenatide + SGLT2i | 575 | 2 | 647 | 0.31 | (0.05-0.90) | (0.05-0.94) |  |
| **Serious Hypoglycemia** | Exenatide, No SGLT2i | 575 | 11 | 853 | 1.29 | 0.65 | 0.70 | 0.49 |
|  | Exenatide + SGLT2i | 575 | 6 | 691 | 0.87 | (0.24-1.78) | (0.26-1.92) |  |

Participants starting open-label GLP-1 RA before matching in exenatide cohort (n=10) and combination cohort (n=26) were censored at time zero. Participants starting open-label GLP-1 RA at or after matching in exenatide cohort (n=19) and combination cohort (n=26) were censored upon initiation of GLP-1 RA (including exenatide) other than study drug.

MACE, major adverse cardiovascular events; SGLT2i, sodium-glucose co-transporter-2 inhibitor. **^†^**Adjustment for: duration of diabetes, age, sex, history of CVD, prior heart failure, prior microalbuminuria, prior macroalbuminuria, baseline eGFR, and baseline HbA_1c_.

**Supplemental Figures**


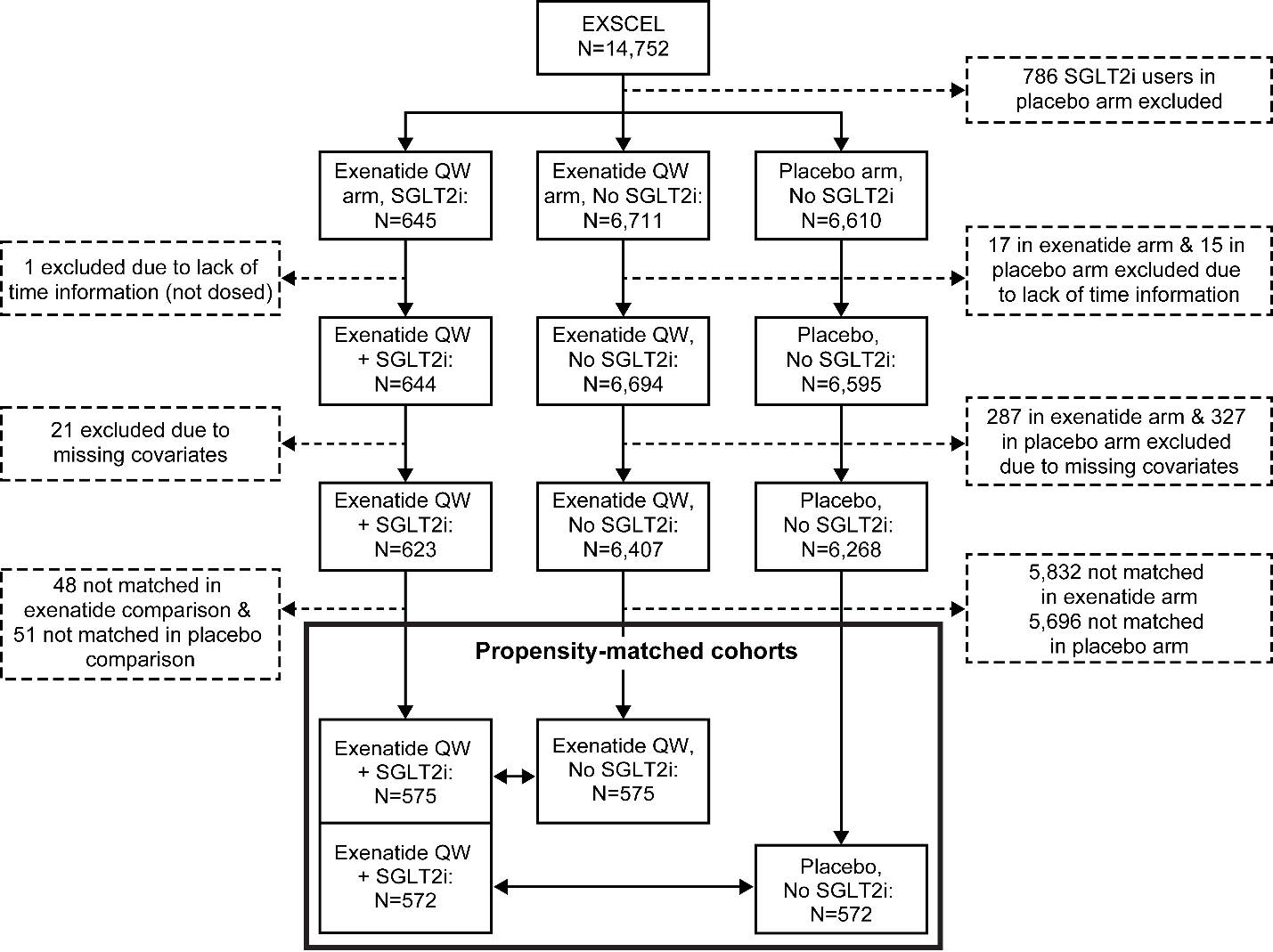


**Figure S1.** Participant flow chart.

Participants were excluded from analysis if they were randomized but not dosed. Participants were excluded from matching if covariates required for matching were missing (no available measurements prior to time of SGLT2i initiation/matching). A large number of non-SGLT2i users were excluded from the propensity-matched cohorts due to the 1:1 matching ratio. SGLT2i, sodium-glucose co-transporter-2 inhibitor.

**
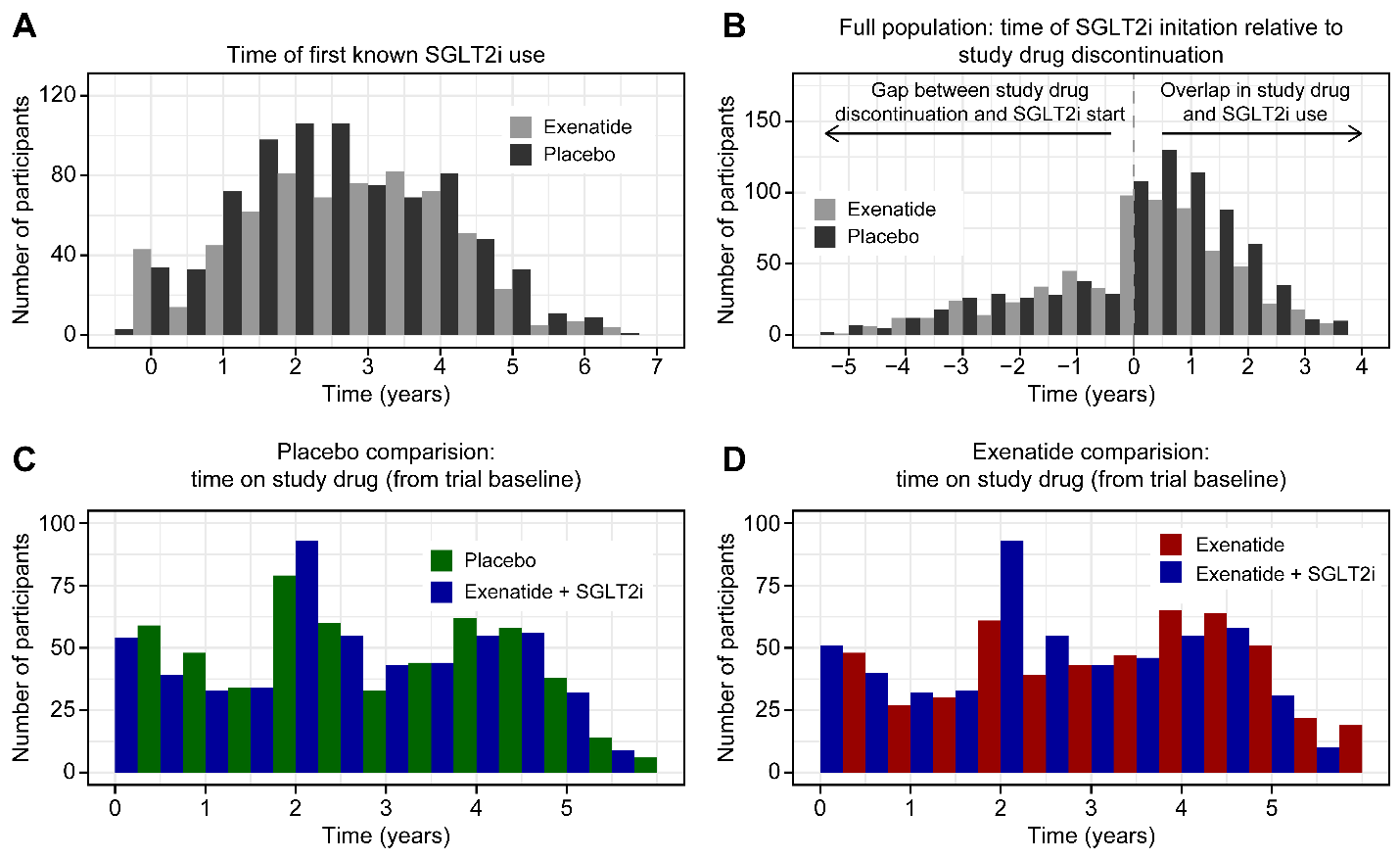
**

**Figure S2.** SGLT2i and study drug usage details.

A) Timing of SGLT2i initiation relative to study baseline in EXSCEL. B) Time of SGLT2i initiation (or matching) relative to discontinuation of exenatide QW or placebo in the full EXSCEL population. C-D) Exenatide QW or placebo treatment time (from trial baseline) in the propensity-matched cohorts. In the placebo comparison, median [IQR] time on study drug from trial baseline was 29.7 months [16.6-48.8] in the placebo cohort and 30.3 months [18.3-48.3] in the combination arm. Median [IQR] time on study drug from trial baseline in the propensity matched cohorts in the exenatide comparison was: 28.4 months [18.2-53.0] in the exenatide cohort and 30.9 months [19.2-49.5] combination cohort. Light gray: exenatide arm, dark gray: placebo arm; blue: combination exenatide + SGLT2i cohorts; green: placebo cohort; red: exenatide cohort.








**Figure S3.** Balance of confounders before (gray) and after (black) propensity matching.

A) Placebo comparison. B) Exenatide comparison. C) Balance between two control cohorts (exenatide and placebo cohorts). Shown as absolute value of standardized difference. A standardized difference of less than 10% (dashed line) was required for all covariates to accept match. p-values in a chi-squared omnibus test for balance were 0.999 for the exenatide comparison, 0.847 for the placebo comparison, and 0.431 between the two control cohorts. Gray = before matching; black = after matching. CV, cardiovascular; DPP4i, dipeptidyl peptidase-4 inhibitor; eGFR, estimated glomerular filtration rate; RAASi, renin-angiotensin-aldosterone system inhibitors; SGLT2i, sodium-glucose co-transporter-2 inhibitor; TZD, thiazolidinedione.





**Figure S4.** Propensity score distributions before and after matching.

A-B) Propensity scores before matching in the placebo (A) and exenatide (B) comparisons. C-D) Propensity scores after matching in the placebo (C) and exenatide (D) comparisons. In each plot, controls are on the left and exenatide + SGLT2i users on the right. Participants with missing covariates required for matching are excluded. SGLT2i, sodium-glucose co-transporter-2 inhibitor.


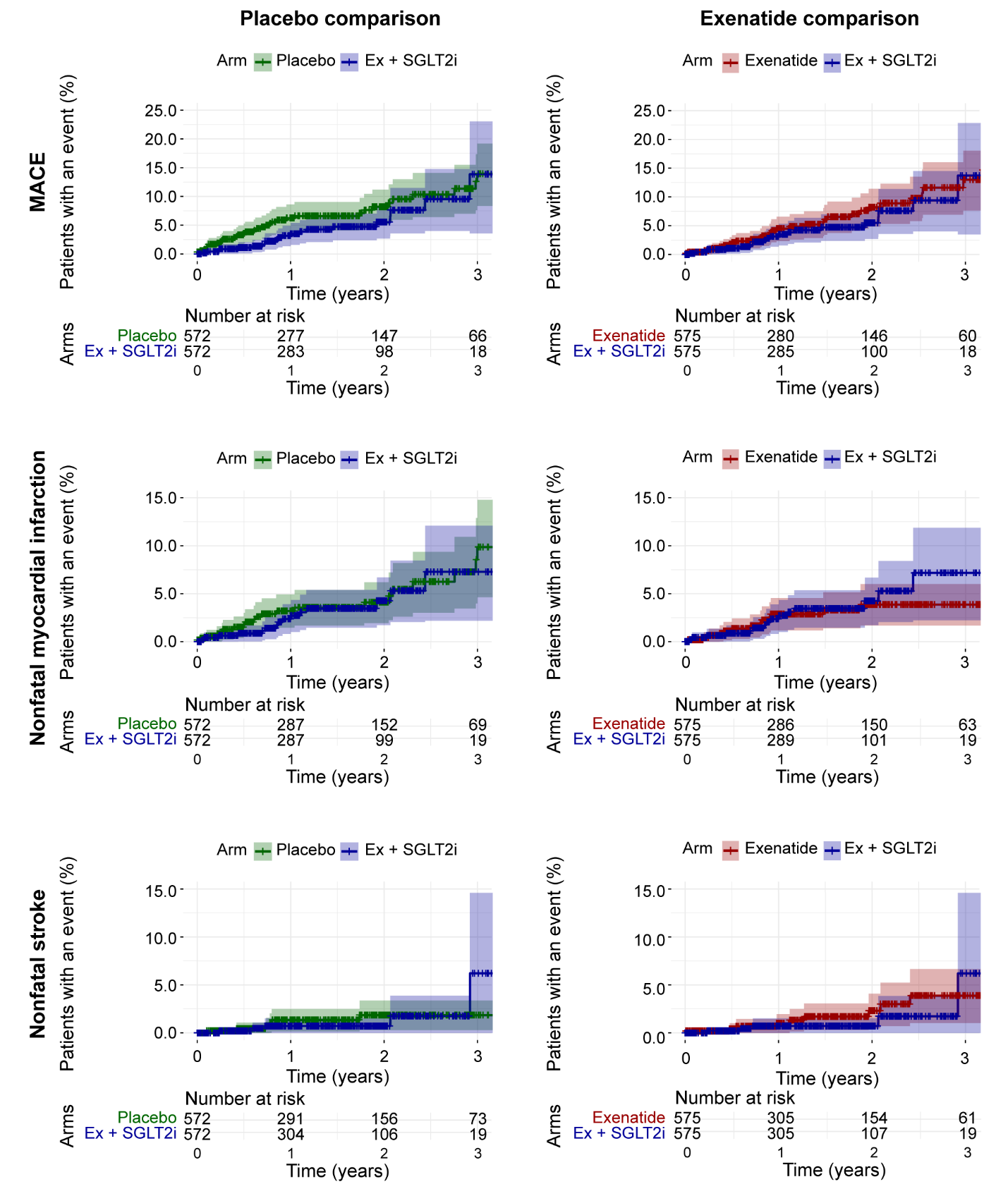
**Figure S5.** Kaplan-Meier curves for MACE, nonfatal MI, and nonfatal stroke.

Shown as percentage of participants with an event. Left: placebo comparison; right: exenatide comparison. Blue: combination exenatide + SGLT2i cohorts; green: placebo cohort; red: exenatide cohort. Crosses indicate events or censoring. Ex, exenatide once weekly; MACE, major adverse cardiovascular events; MI, myocardial infarction; SGLT2i, sodium-glucose co-transporter-2 inhibitor.

**
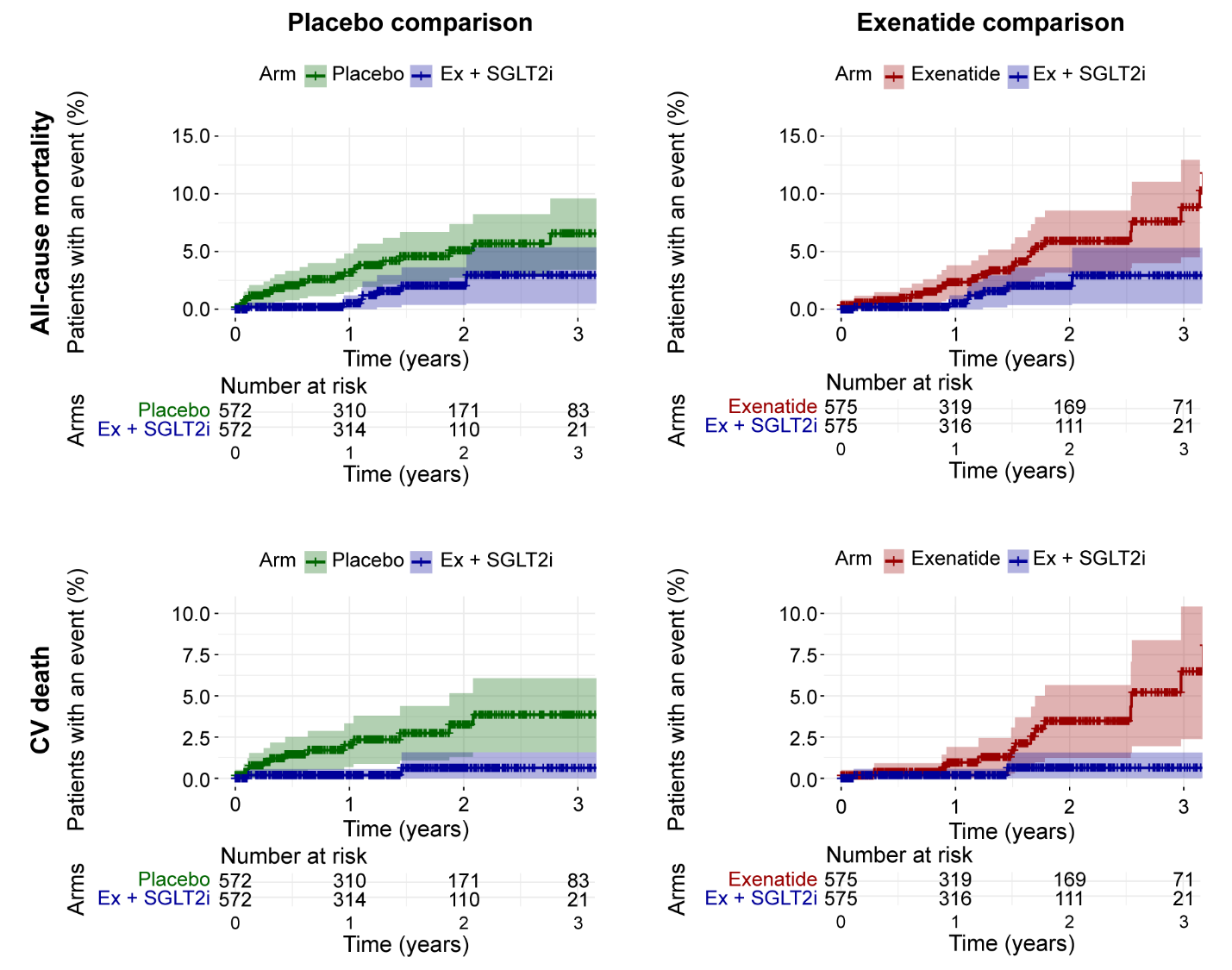
Figure S6.** Kaplan-Meier curves for ACM and CV death.

Shown as percentage of participants with an event. Left: placebo comparison; right: exenatide comparison. Blue: combination exenatide + SGLT2i cohorts; green: placebo cohort; red: exenatide cohort. Crosses indicate events or censoring. Ex, exenatide once weekly; ACM, all-cause mortality; CV, cardiovascular; SGLT2i, sodium-glucose co-transporter-2 inhibitor.

**
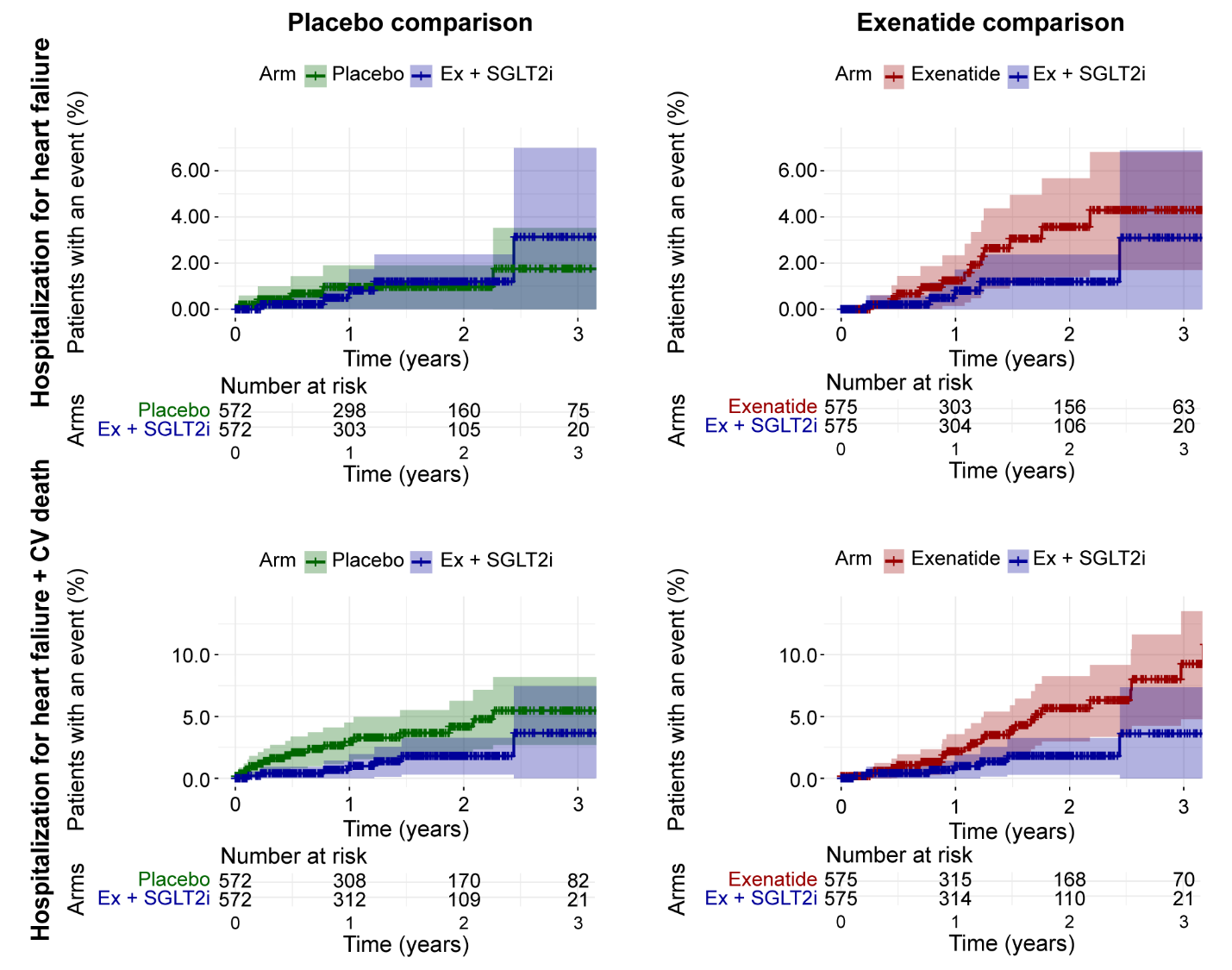
Figure S7.** Kaplan-Meier curves for HHF and HHF + CV death.

Shown as percentage of participants with an event. Left: placebo comparison; right: exenatide comparison. Blue: combination exenatide + SGLT2i cohorts; green: placebo cohort; red: exenatide cohort. Crosses indicate events or censoring. Ex, exenatide once weekly; CV, cardiovascular; SGLT2i, sodium-glucose co-transporter-2 inhibitor.

**
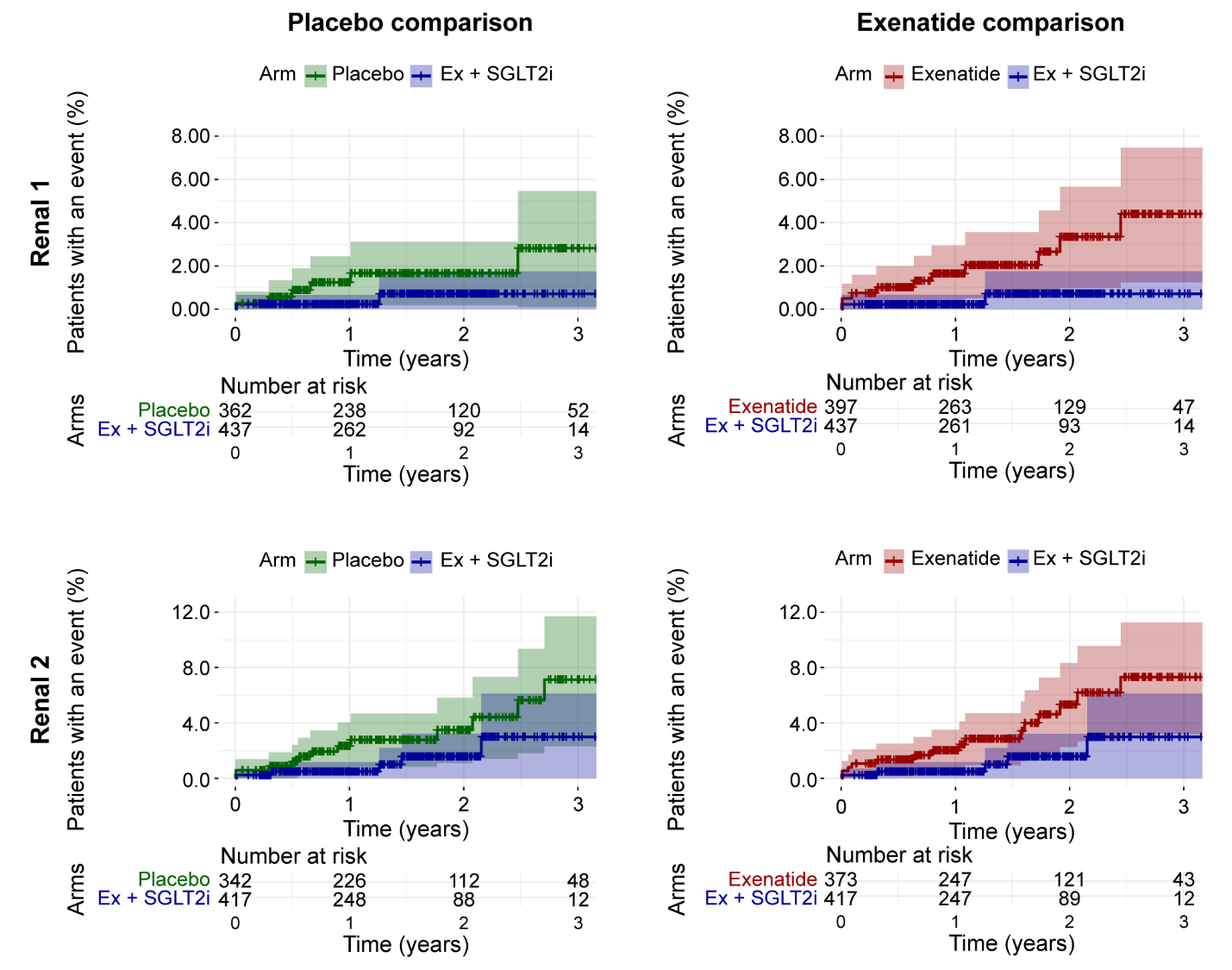
Figure S8.** Kaplan-Meier curves for renal composites.

Shown as percentage of participants with an event. Left: placebo comparison; right: exenatide comparison. Blue: combination exenatide + SGLT2i cohorts; green: placebo cohort; red: exenatide cohort. Crosses indicate events or censoring. Ex, exenatide once weekly; SGLT2i, sodium-glucose co-transporter-2 inhibitor; Renal_1: a composite of a persistent 40% reduction in eGFR, renal dialysis, or renal transplant in participants with 2 or more eGFR measurements after matching; Renal_2: a composite of “Renal 1” and new macroalbuminuria in participants without macroalbuminuria at the time of matching; eGFR: estimated glomerular filtration rate.

**
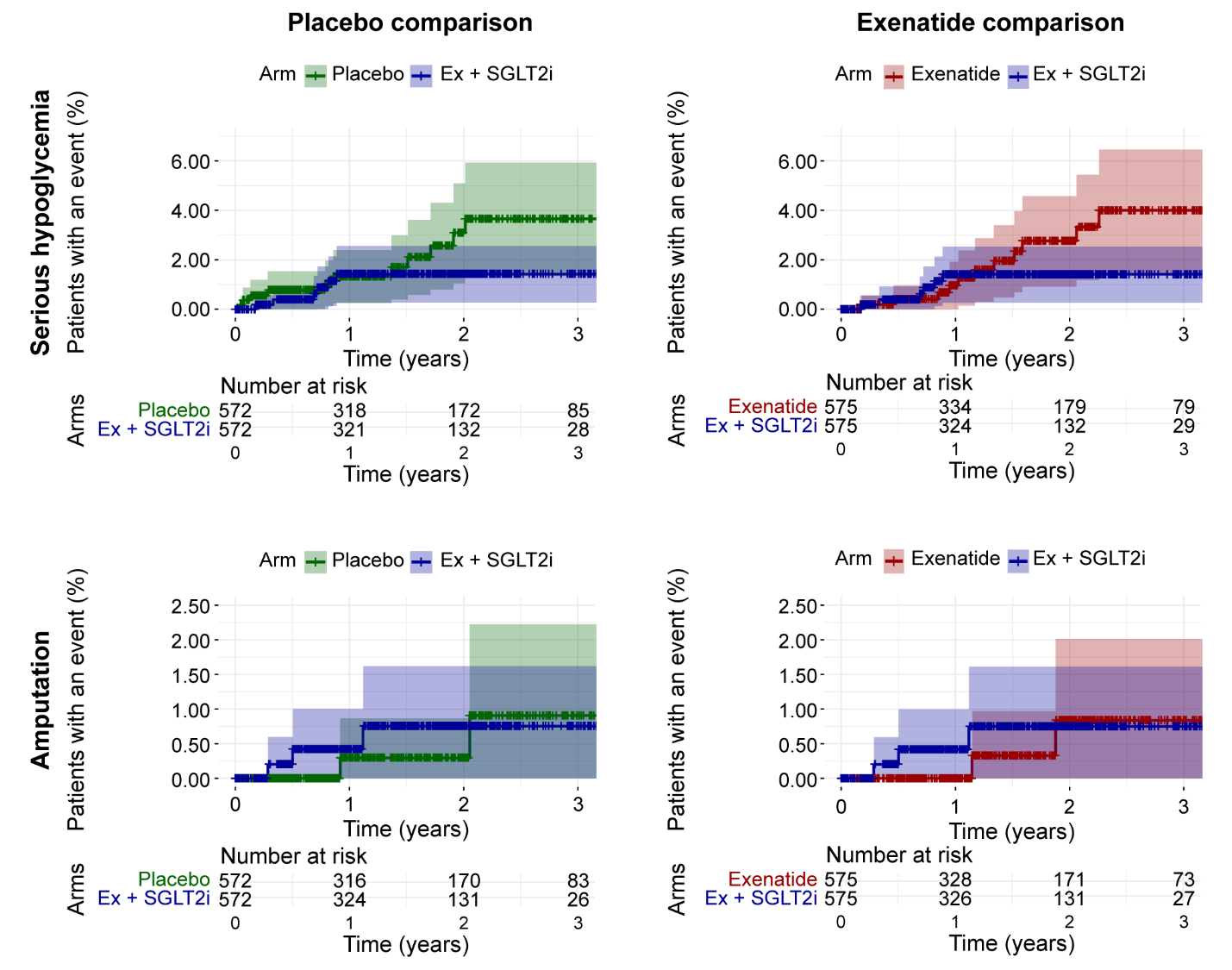
Figure S9.** Kaplan-Meier curves for serious hypoglycemia and amputation.

Shown as percentage of participants with an event. Left: placebo comparison; right: exenatide comparison. Blue: combination exenatide + SGLT2i cohorts; green: placebo cohort; red: exenatide cohort. Crosses indicate events or censoring. Ex, exenatide once weekly; SGLT2i, sodium-glucose co-transporter-2 inhibitor.


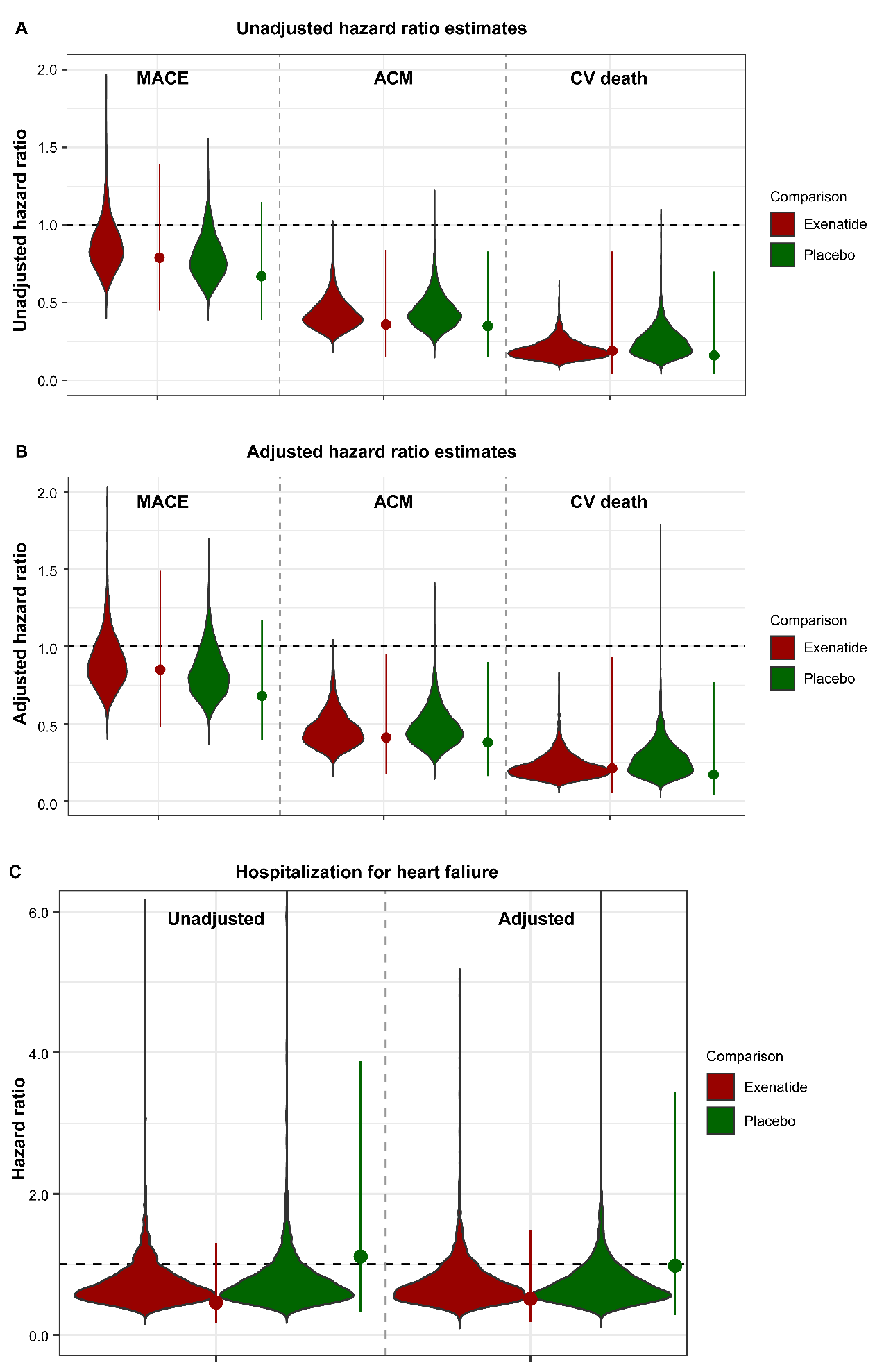


**Figure S10.** Distributions of hazard ratio estimates when matching is repeated.

Hazard ratio distributions in bootstrapped cases (violin plot) compared to the estimated hazard ratio (dot) and 95% confidence interval (line range) in the main analysis case. Matching was repeated 5000 times for participants initiating an SGLT2i between trial baseline and the month 66 visit window, resulting in 1954 accepted cases for the placebo comparison and 2069 accepted cases for the exenatide comparison. A) Unadjusted hazard ratios for MACE, ACM, and CV death. B) Adjusted hazard ratios for MACE, ACM, and CV death. C) Unadjusted (left) and adjusted (right) hazard ratios for hospitalization for heart failure. Note that main analysis hazard ratio for the placebo comparison is higher than the center of the distribution of estimated hazard ratios for hospitalization for heart failure. Green: placebo comparison; red: exenatide comparison. Dashed line shown at a hazard ratio of 1.0. MACE: major adverse cardiovascular events; ACM: all-cause mortality; CV: cardiovascular; SGLT2i.


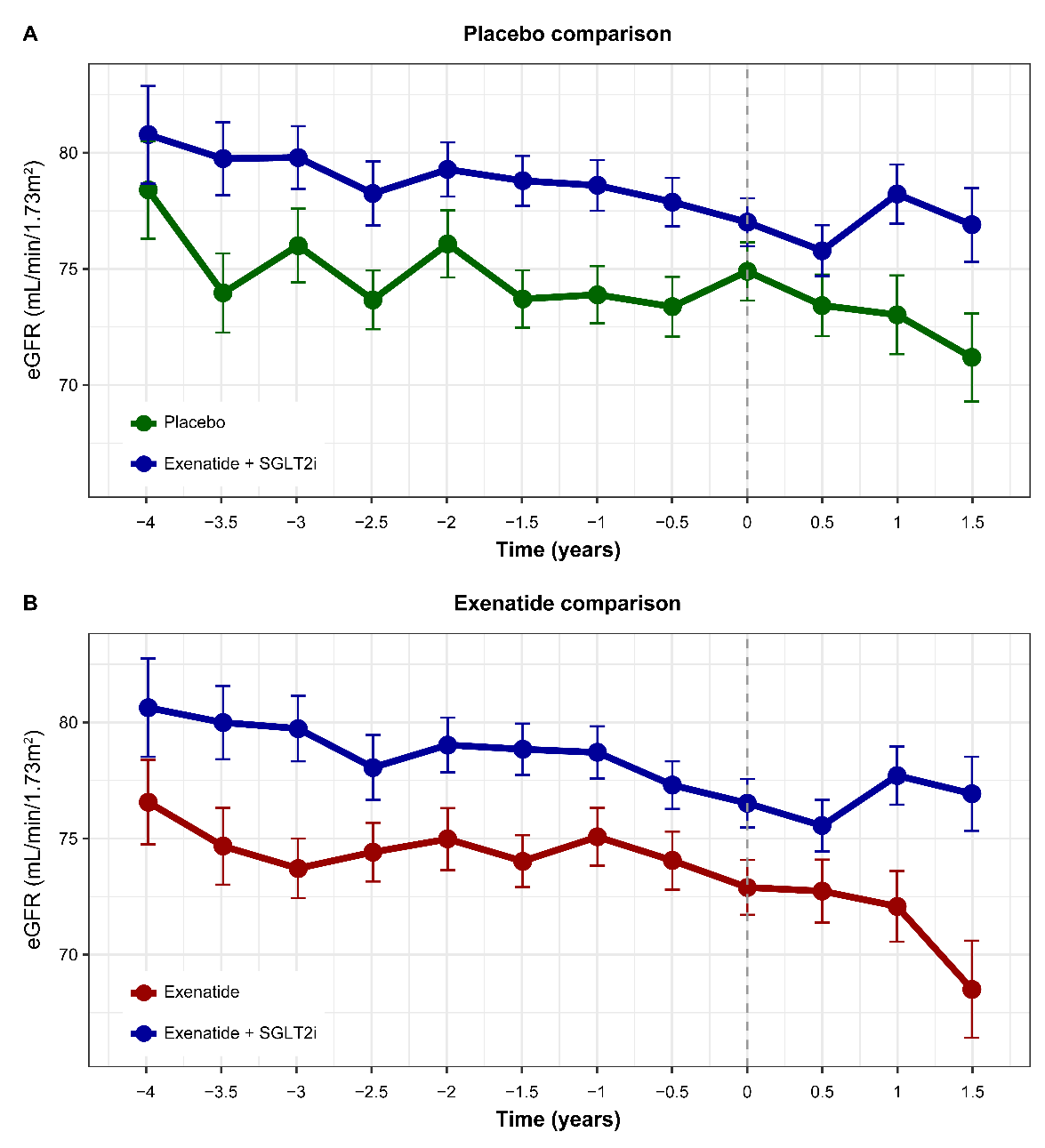


**Figure S11.** Geometric mean (+/- standard error) eGFR slope before and after matching in the propensity-matched cohorts. A) Placebo comparison. B) Exenatide comparison.

MMRM-estimated slopes in the placebo comparison *prior to matching* were: -0.72 mL/min/1.73m^2^/year (standard error 0.20) in the placebo cohort and -0.85 mL/min/1.73m^2^/year (se 0.20) in the exenatide+SGLT2i cohort. In the exenatide comparison, estimated slopes were: -0.27 mL/min/1.73m^2^/year (se 0.19) in the exenatide cohort and -0.79 mL/min/1.73m^2^/year (se 0.19) in the exenatide+SGLT2i cohort.

Time zero is the time of matching; negative times represent time in trial prior to matching, and positive times after matching. Time shown in 6 month visit widows. Note that in **Figure 3**, only times>0 were included in the t=0 window. Blue: combination exenatide + SGLT2i cohorts; green: placebo cohort; red: exenatide cohort. eGFR, estimated glomerular filtration rate; SGLT2i, sodium-glucose co-transporter-2 inhibitor.
